# Focal adhesion kinase inhibitor TAE226 combined with Sorafenib slows down hepatocellular carcinoma by multiple epigenetic effects

**DOI:** 10.1186/s13046-021-02154-8

**Published:** 2021-11-16

**Authors:** Ilaria Romito, Manuela Porru, Maria Rita Braghini, Luca Pompili, Nadia Panera, Annalisa Crudele, Daniela Gnani, Cristiano De Stefanis, Marco Scarsella, Silvia Pomella, Stefano Levi Mortera, Emmanuel de Billy, Adrian Libenzio Conti, Valeria Marzano, Lorenza Putignani, Manlio Vinciguerra, Clara Balsano, Anna Pastore, Rossella Rota, Marco Tartaglia, Carlo Leonetti, Anna Alisi

**Affiliations:** 1 Unit of Molecular Genetics of Complex Phenotypes, Bambino Gesù Children’s Hospital, IRCCS, Via S. Paolo, 15, 00146 Rome, Italy; 2 Unit of Oncogenomic and Epigenetic, IRCCS Regina Elena National Cancer Institute, Rome, Italy; 3 Experimental Imaging Center, IRCCS San Raffaele Scientific Institute, 20132 Milan, Italy; 4 Core Facilities, Bambino Gesù Children’s Hospital, IRCCS, Rome, Italy; 5 Department of Paediatric Haematology/Oncology and Cellular and Gene Therapy, Bambino Gesù Children’s Hospital, IRCCS, Rome, Italy; 6 Unit of Human Microbiome, Multimodal Laboratory Medicine Research Area, Bambino Gesù Children’s Hospital, IRCCS, Rome, Italy; 7 Unit of Microbiomics, Microbiology and Immunological Diagnostics, Department of Diagnostics and Laboratory Medicine Bambino Gesù Children’s Hospital, IRCCS, Rome, Italy; 8 International Clinical Research Center, St. Anne’s University Hospital, Brno, Czech Republic; 9 Department of Translational Stem Cell Biology, Research Institute of the Medical University of Varna, 9002 Varna, Bulgaria; 10 Department of Life, Health and Environmental Sciences MESVA, University of L’Aquila, L’Aquila, Italy; 11 Francesco Balsano Foundation, Rome, Italy; 12 Research Unit of Diagnostical and Management Innovations, Bambino Gesù Children’s Hospital, IRCCS, Rome, Italy; 13 Genetics and Rare Diseases Research Division, Bambino Gesù Children’s Hospital, IRCCS, Rome, Italy

**Keywords:** HCC, FAK, Sorafenib, Epigenetic, Therapy

## Abstract

**Background:**

Hepatocellular carcinoma (HCC) is one of the most common and lethal malignant tumours worldwide. Sorafenib (SOR) is one of the most effective single-drug systemic therapy against advanced HCC, but the identification of novel combination regimens for a continued improvement in overall survival is a big challenge. Recent studies highlighted the crucial role of focal adhesion kinase (FAK) in HCC growth. The aim of this study was to investigate the antitumor effects of three different FAK inhibitors (FAKi), alone or in combination with SOR, using in vitro and in vivo models of HCC.

**Methods:**

The effect of PND1186, PF431396, TAE226 on cell viability was compared to SOR. Among them TAE226, emerging as the most effective FAKi, was tested alone or in combination with SOR using 2D/3D human HCC cell line cultures and HCC xenograft murine models. The mechanisms of action were assessed by gene/protein expression and imaging approaches, combined with high-throughput methods.

**Results:**

TAE226 was the more effective FAKi to be combined with SOR against HCC. Combined TAE226 and SOR treatment reduced HCC growth both in vitro and in vivo by affecting tumour-promoting gene expression and inducing epigenetic changes via dysregulation of FAK nuclear interactome. We characterized a novel nuclear functional interaction between FAK and the NuRD complex. TAE226-mediated FAK depletion and SOR-promoted MAPK down-modulation caused a decrease in the nuclear amount of HDAC1/2 and a consequent increase of the histone H3 lysine 27 acetylation, thus counteracting histone H3 lysine 27 trimethylation.

**Conclusions:**

Altogether, our findings provide the first evidence that TAE226 combined with SOR efficiently reduces HCC growth in vitro and in vivo. Also, our data highlight that deep analysis of FAK nuclear interactome may lead to the identification of new promising targets for HCC therapy.

**Supplementary Information:**

The online version contains supplementary material available at 10.1186/s13046-021-02154-8.

## Background

Hepatocellular carcinoma (HCC) has been recognized as the fifth most common type of cancer, accounting for 70 to 85% of liver cancers, with an estimated 5-year survival less than 20% [1]. HCC is notoriously resistant to systemic therapies, and often recurs after aggressive local therapies [2]. Currently, systemic therapies have challenged the use of conventional therapies for HCC. Sorafenib (SOR), an orally available multi-kinase inhibitor, is one of the most effective single-drug systemic therapy and numerous clinical studies have shown that this drug provides good survival benefits in patients with advanced HCC [3–5].

However, SOR treatment causes several off-target and side effects, and response is transient due the occurrence of resistance, which is generally reported within 6 months of treatment. Several mechanisms have been implicated in the reduction of tumour cell sensitivity to SOR, including epigenetic, transport processes, regulated cell death, and the tumour microenvironment [6].

The partial success of SOR has generated enthusiasm in the development of new molecules to be used as first- or second-line agents for systemic treatment of HCC. Between 2017 and 2018, four drugs (Lenvatinib, Regorafenib, Cabozantinib, and Ramucirumab), were found to be effective and tolerated, and have been approved by the Food and Drug Administration (FDA) as first- or second-line therapeutic agents for HCC patients [4, 5, 7, 8]. To minimize resistance that often occurs as a consequence of the multi-step nature of HCC pathogenesis, the use of multi-target and/or combined therapies are suggested as the optimal strategy. Indeed, Kudo et al. have recently reported the efficacy of combining transarterial chemoembolization (TACE) with SOR [9]. Therefore, considering that SOR currently remains the first-line agent for the treatment of unresectable HCC, it is imperative to identify new targetable molecules able to impair SOR resistance by investigating signalling pathways that are dysregulated in HCC onset and progression.

Recently, studies highlighted that focal adhesion kinase (FAK), often activated by autophosphorylation at Tyr-397 (pTyr397FAK) and over-expressed in liver cancer, could be one potential druggable target to fight HCC [10–12]. Accordingly, Gnani et al. [13] showed that FAK depletion reduces HCC cell growth by affecting cancer-promoting genes, including the pro-oncogene Enhancer of zeste homolog 2 (EZH2). This study unveiled a previously unappreciated FAK/EZH2 crosstalk in HCC cells, thus identifying a targetable network paving the way for new anticancer therapies. Besides that, it has been suggested that the use of FAK inhibitors (FAKi) in combination with other FDA approved therapies could be a promising therapeutic option for HCC [14]. In particular, PND1186 effectively inhibits Dasatinib induced pTyr397FAK expression, and synergizes with Dasatinib to block HCC cell growth in vitro by decreasing proliferation and inducing apoptosis [14]. Interestingly, Azzariti et al. [15] demonstrated that SOR resistance could be caused by the crosstalk between the tissue microenvironment and HCC through the hepatic stellate cells secreting Ln-332, a major ligand for α3β1 integrin that leads to recovery the ubiquitinated FAK by SOR.

A number of FAKi are commercially available and some of them have been tested in clinical trials in other tumour types, such as glioblastoma and lung cancer [16, 17]. To date, no studies have evaluated the performance of these drugs in reducing HCC cell growth either alone or in combination with SOR.

Here, we investigated the effects of three different FAKi, i.e., PND1186, PF431396 and TAE226, on HCC cells viability. We deeply explored the antitumor effects of TAE226, which emerged as the most effective FAKi, alone or in combination with SOR.

We found that TAE226 combined with SOR efficiently reduced tumour growth in in vitro and in vivo models of HCC by exerting multiple epigenetic effects strongly associated with FAK nuclear interactome.

## Methods

### Cell lines

The human HepG2 cells were purchased from ATCC (Manassas, Virginia, USA) that provided certificated authentication, and previously certified Huh7 cells. All cell lines were grown in Dulbecco’s modified Eagle’s medium supplemented with 10% FBS, 1% L-glutamine, and antibiotics (1% penicillin/streptomycin) at 37 °C in 5% CO_2_ in a 95% humidified atmosphere. All cells used for this study resulted negative to the presence of *Mycoplasma* spp. after testing by Venor GeM Advance Mycoplasma Detection Kit (Minerva Biolabs, Berlin, Deutschland).

To follow in vivo tumour growth, HepG2 cells were stably transfected with an inducible plasmid encoding firefly luciferase and selected with G418 to produce HepG2-Luc cells as already described [13].

### Treatments

SOR, PND1186 and PF431396 (Selleck Chemicals, Houston, TX, USA) and TAE226 (kindly provided by Novartis Pharm) were dissolved in dimethyl sulfoxide and stored at − 80 °C until time of use. HCC cells were exposed to different concentration of drugs for different times as detailed in the results.

Cells were lentivirally silenced for FAK as already described [13].

### Cell viability assay

Cell viability was evaluated by using a colorimetric assay (XTT based) for the non-radioactive quantification of cell proliferation and viability (Roche Molecular Biochemicals, Indianapolis, Indiana, USA), according to the manufacturer’s protocol. The absorbance of the water-soluble formazan formed was measured at 490 nm using the ELISA Benchmark Plus microplate spectrophotometer (Bio-Rad Laboratories, Hercules, California, USA). The viability of the treated cells was expressed as the percentage of killed cells versus untreated cells. The Combination Index (CI) was calculated with the Chou-Talalay method using CalcuSyn v.2.0 software in order to calculate the synergism, additivity or antagonism of the different drug combinations [18].

### Cell proliferation assay

Cells were plated in a 96 microplate well. Next, 5-bromo-2’deoxyuridine (BrdU) assay was performed by using the Dissociation-Enhanced Lanthanide Fluorescent Immunoassay (DELFIA) Cell Proliferation Kit following the manufacturer instructions (Perkin Elmer, Waltham, Massachusetts, USA). The fluorescence, which is proportional to BrdU incorporation during DNA synthesis as a measure of cell proliferation, was determined by time-resolved fluorometer 2100 EnvisionTM Multilabel Reader (Perkin Elmer).

### Cell cycle and apoptosis analysis

Cell cycle phase distribution was analysed by flow cytometry using propidium iodide (PI) staining (Sigma-Aldrich, St. Louis, Missouri, USA). HCC cells were collected by trypsinization, washed with PBS, then fixed in a cold solution of methanol/acetone (4:1). Cells were first incubated with RNase A at 37 °C then stained with a solution containing 100 μg/ml PI, at 37 °C for 20 min. Stained nuclei were analysed for DNA-PI fluorescence using a Becton Dickinson FACSCanto II flow cytometer (Becton-Dickinson, Milan, Italy). The proportions of cells in G0/G1, S phase, and G2/M phases of the cell cycle were analysed by DiVa Software, version 6.3 (Becton-Dickinson).

Apoptosis was assessed by FITC Annexin V Apoptosis Detection Kit (Becton-Dickinson). Briefly, cells were washed in PBS and re-suspended in Annexin Binding Buffer (10 mmol/L HEPES pH 7.4, 140 mmol/L NaCl, and 2.5 mmol/L CaCl). Cells were then stained with 0.5 μl Annexin V-FITC and 5 μl PI (Becton-Dickinson) for 15 min before analysing. Acquisition and analysis were carried out on a Becton Dickinson FACSCanto II flow cytometer, using DiVa Software, version 6.3.

### Western blotting (WB)

Cells were collected and total protein extraction was performed by RIPA lysis buffer (Cell Signaling Technology, Danvers, Massachusetts, USA) containing 1X protease and phosphatase inhibitors cocktail. The homogenates were then centrifuged at 13000 rpm at 4 °C for 10 min and the resulting supernatant was taken as protein sample. Whole cell extracts were quantified using the BCA Protein Assay (Thermo Fisher Scientific Inc., Waltham, Massachusetts, USA). Samples were then diluted in the sample buffer (200 mM Tris-HCl (pH 6.8), 40% glycerol, 20% β-mercaptoethanol, 4% sodium dodecyl sulphate, and bromophenol blue) and resolved in SDS-PAGE, then transferred and immobilized onto nitrocellulose membranes (Amersham, Little Chalfont, UK). The membranes were blocked using 5% non-fat dry milk for 30 min and incubated with the appropriate primary and secondary antibodies. Protein expression was quantified by densitometry analysis using Image J v3.91 software. The used antibodies are listed in Table S1.

### Spheroid generation and image acquisition

200 cells/well of HepG2 and 400 cells/well of Huh7 were seeded in 100 μl of complete growth medium on 96 Ultra-Low Attachment (Corning Life Sciences, Amsterdam, Netherlands) well plates to obtain, 96 h after cell seeding, tumour spheroids (TS) of 250 μm in diameter. Forty-eight hours after treatments, diameters and area were calculated and TS were stained using PI (1 mg/ml final concentration). Image capture and analysis were performed using Celigo imaging cytometer (Nexcelom Biosciences LLC, Lawrence, Kansas, USA).

### Anchorage-independent soft agar colony formation assay

HCC cells were treated as expected from the experiment and then mixed with 0.3% agarose and were plated over a 0.6% agarose layer. The medium was renewed twice weekly. After 21–30 days, the cells were stained with 0.05% crystal violet solution and counted manually. The data are represented as number of colonies per well.

### Animals

Animal procedures were in compliance with the national and international directives (D.L. 4 March 2014, no. 26; directive 2010/63/EU of the European Parliament and of the council; Guide for the Care and Use of Laboratory Animals, United States National Research Council, 2011) and approved by the Italian Ministry of Health (authorization n. 213/2019-PR, released date 03/13/2019).

Six to eight week-old CD1-nude or NOD/SCID male mice were used (Charles River laboratories, Calco, Milan, Italy) to establish xenograft model of HCC as already reported [13]. Briefly, HepG2-Luc were intramuscularly (heterotopic) injected in nude mice at 5 × 10^6^ cells/mice for pilot study or injected into the liver (orthotopic) of NOD/SCID mice at 10^6^ cells/mice. Treatments started when a well-established tumour (400 mg) was evident in mice (day 19 after tumour cells injection) as measured by a caliper in heterotopic tumours, while mice bearing intrahepatic tumours were imaged using the IVIS imaging system 200 series (Perkin Elmer).

In the heterotopic pilot study, 8 CD1-nude mice for each group of treatment were used to identify the adequate concentration of TAE226 for in vivo setting of the drug. Therefore, at day 19, the animals were divided in three groups and treated orally by gavage as follows: Group 1A: Vehicle; Group 2A: TAE226 at 25 mg/kg for two weeks; Group 3A: TAE226 at 50 mg/kg for two weeks.

In order to evaluate the effect of combination therapy, at day 18 the orthotopically injected NOD/SCID mice (*n* = 24) were divided in four groups and treated as follows: Group 1B: Vehicle; Group 2B: TAE226 at 25 mg/kg orally for five consecutive days repeated for 2 weeks; Group 3B: SOR at 30 mg/kg orally for five consecutive days; Group 4B: TAE226 at 25 mg/kg for five consecutive days repeated for 2 weeks followed by SOR at 30 mg/Kg for five consecutive days.

Mice were analysed by imaging at different times before tumour cells injection and during the treatment. At day 35 from the start of treatments the animals were sacrificed. Imaging data were acquired at different timepoints (days from treatment 0, 14, 21, 28 and 35) and analysed using the Living Image Software version 4.3 (Perkin Elmer).

### Immunofluorescence

Immunofluorescence was performed on 2 μm-thick sections obtained from formalin-fixed tissue embedded in paraffin. Antigen retrieval was performed with EDTA (pH 8) (Dako, Glostrup, Denmark). The sections were incubated overnight with specific primary antibodies at 4 °C (Table S1).

The primary antibodies were revealed with the secondary antibodies purchased by Alexa Fluor (Thermo Fisher Scientific Inc.). The confocal microscopy imaging was performed on Olympus Fluoview FV1000 confocal microscope equipped with FV10-ASW version 2.0 software, using 20X and 40X objective. Fluorochromes unmixing was performed by acquisition of automated-sequential collection of multi-channel images, in order to reduce spectral crosstalk between channels. The sections were incubated overnight with specific primary antibodies at 4 °C.

### Gene expression and pathways enrichment

A pre-designed TaqMan OpenArray Human Cancer Panel (Thermo Fisher Scientific Inc.) was used to assess the effect of FAK inhibition plus SOR in HepG2 and Huh7 cells on a signature panel of 624 well-defined genes validated for the characterization of cancers, plus 24 endogenous controls. cDNAs were loaded onto the Open Array platform and run as recommended by the manufacturer on the QuantStudio 12 K Flex Real-Time PCR system (Thermo Fisher Scientific Inc.). Relative gene expression values were calculated as relative quantity (RQ) by using Open Source expression suite provided by Life Technologies (Thermo Fisher Scientific Inc.). RQ minimum and maximum values were calculated with a confidence level of 95%, using Benjamini-Hochberg false discovery rate (FDR) to adjust *p* values. Maximum allowed Ct included in calculations were 35.

Pathway analysis was conducted by querying Reactome annotations using the R/Bioconductor library reactome.db [19, 20]. For Reactome analysis, only pathways with a FDR lower than 0.05 and *p* < 0.05 were considered.

### Detection of histone H3 lysine 27 trimethylation

HepG2 and Huh7 cells were cultured and then treated with the combined therapy or with the single inhibitors. At the end of the treatment, AlphaLISA assay was conducted using the Tri-Methyl-Histone H3 Lysine 27 (H3K27me3) Cellular Detection Kit (Perkin Elmer) following the manufacturer’s instructions. Detection was performed with an EnVision Multilabel Reader (Perkin Elmer) using the AlphaScreen standard settings as previously described [21].

### Cell imaging

Cells were cultured in a 96 well collagen coated plate (Perkin Elmer) or in 4-well chamber slides and treated with combined therapy TAE226 plus SOR. Next, cells were fixed with a solution of methanol/acetone (3:1) at − 20 °C for 20 min. After two brief washes with PBS, cells were blocked with PBS/BSA 1% at room temperature (RT) for 30 min and then incubated with primary antibodies (Table S1) diluted in PBS/BSA 1% for 2 h at RT. Then, cells were washed twice with PBS and incubated with 1:500 Alexa Fluor 488 goat anti-rabbit IgG secondary antibody (Thermo Fisher Scientific Inc.) in PBS/BSA 1% for 30 min at RT. Next, cells were washed with PBS and then incubated with 1:15000 Hoechst in PBS/BSA 1% for 5 min at RT for nuclear staining.

Cell imaging was performed by Operetta CLS in confocal mode (Perkin Elmer) and the 40X water immersion objective. Image segmentation and analysis were performed with the Harmony software 4.8 (Perkin Elmer) for the determination of the mean intensity by wells of the fluorescence signal.

Otherwise imaging was performed by using the original digital images format acquired with an Olympus FV3000 confocal microscope. For quantification of expression, *n* = 4 randomly selected images per each cell line in two independent experiments were used. The Mean Fluorescence Intensity was analysed using Olympus CellSens Dimension Desktop 2.3.

### Nuclear protein extraction

For extraction of nuclear protein, cells were washed with PBS, trypsinized, pelleted by centrifugation at 1200 rpm for 5 min and then resuspended in the cell lysis buffer [10 mM HEPES pH 7.5, 10 mM KCl, 0.1 mM EDTA, 1 mM dithiothreitol (DTT), 0.5% Nonidet-40 and 0.5 mM PMSF along with the 1x protease and phosphatase inhibitor cocktail (Thermo Fisher Scientific Inc.)] and kept in ice for 15–20 min with intermittent mixing. Then, tubes have been vortexed to disrupt cell membranes and centrifuged at 12000 g at 4 °C for 10 min.

The pelleted nuclei were washed with the cell lysis buffer, resuspended in the nuclear extraction buffer [20 mM HEPES pH 7.5, 400 mM NaCl, 1 mM EDTA, 1 mM DTT, 1 mM PMSF with 1x protease and phosphatase inhibitor cocktail] and then incubated in ice for 30 min. Nuclear extract was collected by centrifugation at 12000 g for 15 min at 4 °C. Protein concentration was estimated using BCA Protein Assay. The extract is either immediately used or stored at − 80 °C until further use.

### Immunoprecipitation

Immunoprecipitation samples (500 μg nuclei) were pre-cleared with protein A/G PLUS-Agarose beads (Santa Cruz Biotechnology, Dallas, Texas, USA) for 2 h at 4 °C under end-to-end rotation. At the end of the incubation, the samples were washed with TBS, centrifuged for 30 s at 5000 g at RT, and supernatant was recovered. Next, 20 μl of conjugates between beads and primary antibody (Table S1) were added to supernatant and incubated for 2 h at 4 °C under end-to-end rotation. Negative controls (Mock) were performed with protein A/G PLUS-Agarose beads alone (without adding antibody to the extract). At the end of the incubation, the beads were centrifuged for 30 s at 5000 g at RT, and supernatant was discarded. Then, the immunoprecipitates were washed three times by adding 200 μl of TBS, were eluted in sample buffer and resolved by 12% SDS-PAGE.

### Proteomic analysis

Immunoprecipitates were separated by mono-dimensional SDS-PAGE and resolved proteins were visualized with QC Colloidal Coomassie stain (Bio-Rad Laboratories). Gel lanes were excised into 10 bands and in-gel digested [22]. Extracted peptides were subjected to nanoLiquid Chromatography-ElectroSpray Ionization-tandem Mass Spectrometry (nLC-ESI-MS/MS) analysis performed on an UltiMate3000 RSLCnano System directly coupled to the Orbitrap Fusion Tribrid mass spectrometer (Thermo Fisher Scientific Inc.). The peptide mixtures were first trapped and desalted onto a μ-precolumn C18 PepMap100 (5 μm particle size, 100 Å pore size, 300 μm i.d. × 5 mm length, Thermo Fisher Scientific Inc.) for 3 min at 10 μL/minutes with an aqueous solution of 2% acetonitrile and 0.1% trifluoroacetic acid, and then separated by reverse-phase chromatography performed on an EASY-Spray PepMap RSLC C18 column (2 μm particle size, 100 Å pore size, 75 μm i.d. × 50 cm length, Thermo Fisher Scientific Inc.) at a flow rate of 250 nL/minutes, at a temperature of 35 °C, by a one-step linear gradient starting from 98% eluent A (0.1% formic acid, FA, in water) to 25% eluent B (99.9% ACN, 0.1% FA) in 60 min, and a total LC-run of 96 min. Orbitrap detection was used for precursor (MS1) ions measurements at resolving powers of 120 K (at 200 m/z), whereas fragments (MS2, MS/MS) ions were recorded by Ion Trap at rapid scan rate. Data dependent MS/MS analysis was performed in top speed mode with a 3 s cycle-time, during which most abundant multiple-charged (2+ − 7+) precursor ions detected within the range of 250–1500 m/z were selected for activation in order of abundance. The signal intensity threshold for MS2 was 5 × 10^3^. Quadrupole isolation with a 1.6 m/z isolation window was used, and dynamic exclusion was enabled for 1 min. High-energy collisional dissociation was performed using 30% normalized collision energy. Automatic gain control targets were 4.0 × 10^5^ for MS and 2 × 10^3^ for MS2, with 50 and 300 ms maximum injection times, respectively. The option “Injection Ions for All Available Parallelizable Time” was set.

Proteins were identified with the SequestHT algorithm embedded in the Proteome Discoverer software (version 1.4, Thermo Fisher Scientific Inc.) by merging the 10 raw data files for each lane in a single analysis and interrogating the *Homo sapiens* UniProtKB reference proteome (ID: UP000005640, release: 2020_03, 20,621 sequence entries). The search parameters were set to use a tolerance of 10 ppm and 0.6 Da for precursor ions and product ions respectively, allowing 1 missed cleavage. Carbamidomethylation of cysteine was set as fixed modification while oxidation of methionine, phosphorylation of serine, tyrosine and threonine, and protein N-terminal acetylation were chosen as variable modifications.

A FDR threshold of 0.01, using Percolator algorithm for PSM validation, was used for the identifications

Results were filtered in all experiments considering only those identified proteins with at least two unique peptides and a SumPEP score value ≥50. Keratines were also filtered out from the resulting protein list.

### Analysis of protein-protein interaction (PPI) networks

The STRING (Search Tool for Recurring Instances of Neighbouring Genes) database [23] was used for characterization of nuclear interactors of FAK. PPI networks between nuclear interactors of FAK were identified by typing proteins names in the software along with the selection of the species under investigation (*H. sapiens*). In order to exclude most of the false positive interactions as possible, we used 0.7 (high level of confidence) as the total STRING protein interaction confidence scores and six number of clusters for PPI filtering. Gene Ontology (GO) enrichment analysis was carried out by using STRING online tools.

### Detection of histone H3 lysine 27 acetylation

AlphaLISA assay was conducted similarly to the H3K27me3 analysis by using the AlphaLISA Acetylated-Histone H3 Lysine 27 (H3K27ac) Cellular Detection Kit (Perkin Elmer).

### Statistics

Multivariate Student’s t-test or one-way ANOVA were applied and values of *p* < 0.05 were considered statistically significant. GraphPad Prism 5.0 was used for statistical analysis.

## Results

### Effects of FAKi on cell viability, cell proliferation and FAK autophosphorylation in HCC cells

**Fig. 1:**
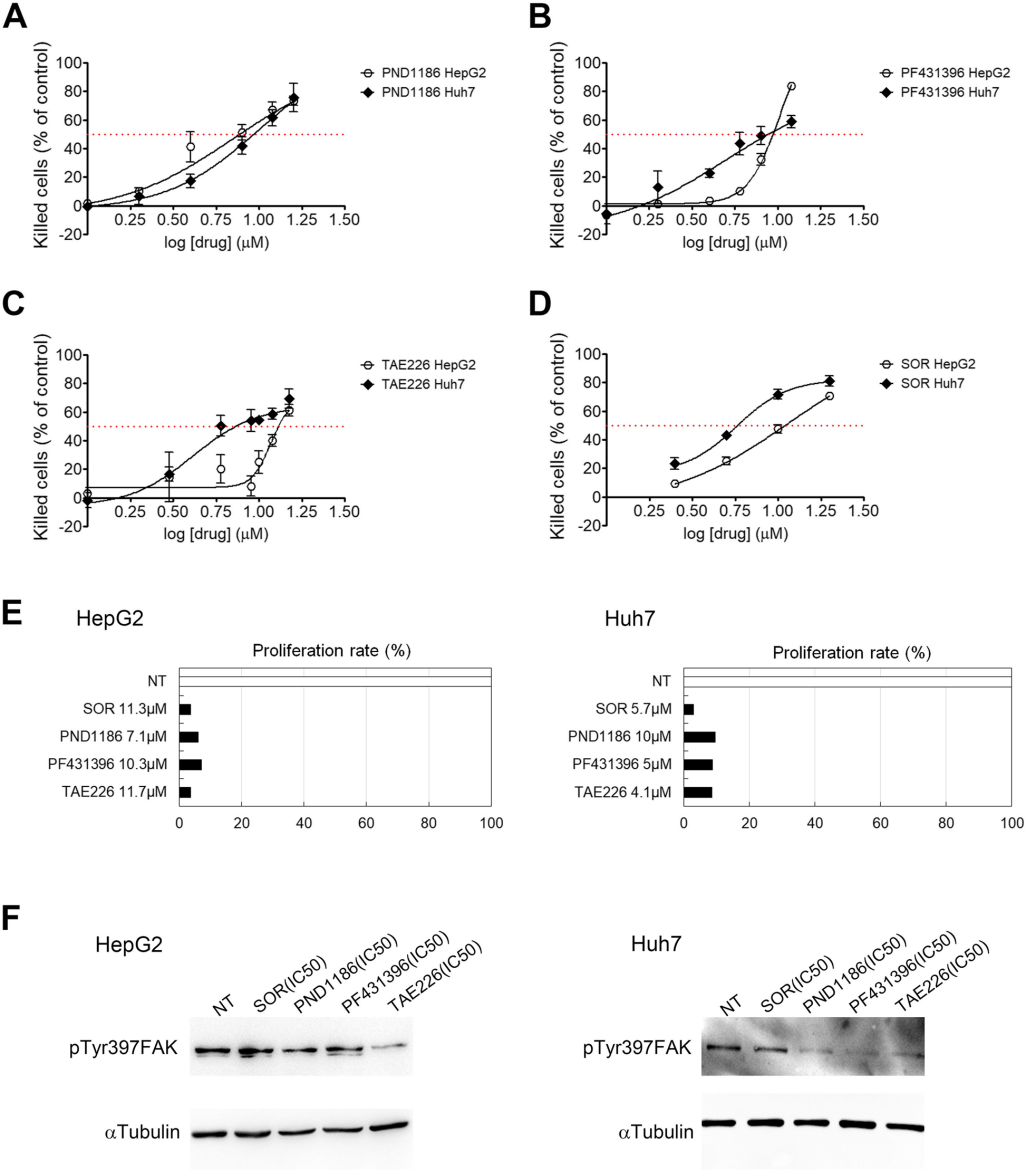
Effect of drugs on cell viability, cell proliferation and FAK phosphorylation in HCC cells. Dose-response curves and IC50 fit lane for PND1886 (**A**), PF431396 (**B**), TAE226 (**C**), and SOR (**D**) in HepG2 and Huh7 cells. Values are measured by XTT assay after 48 h from treatment and expressed as percentage of killed cells respect to NT ± Standard Deviation (SD) of three independent experiments. **E** Proliferation rate in HCC cells after treatment with IC50 values in HepG2 and Huh7 cells. Values are expressed as percentage respect to NT cells of the mean of at least three independent experiments. **F** Representative immunoblot by WB of pTyr397FAK expression after 48 h from treatment with the different drugs, in HepG2 and Huh7 cells. αTubulin served as loading control

We first looked at the sensitivity of HepG2 and Huh7 HCC cell lines to three different commercially available FAKi (PND1186, PF431396, TAE226) and to SOR treatments. We performed a dose response assay to determine the half-maximal inhibitory concentration values (IC50) following 48 h of treatment. As shown in Fig. 1A-D, all compounds reduced cell viability evaluated as percentage of killed cells with respect to non-treated (NT) cells. In particular, the following values of IC50 were determined: PND1186 was 7.1 μM for HepG2 cells and 10 μM for Huh7 cells (Fig. 1A); PF431396 was 10.3 μM for HepG2 cells and 5 μM for Huh7 cells (Fig. 1B); TAE226 was 11.7 μM for HepG2 cells and 4.1 μM for Huh7 cells (Fig. 1C); SOR was 11.3 μM for HepG2 cells and 5.7 μM for Huh7 cells (Fig. 1D). Then, we evaluated cell proliferation rate following 48 h of treatment with the different drugs at the IC50 concentrations obtained for both cell lines. From this analysis, SOR and TAE226 resulted as the most efficient in reducing cell proliferation in HepG2 cells, while SOR proved to be the most effective treatment, followed by PF431396 and TAE226 in Huh7 cells.

As shown in Fig. 1F and Fig. S1, TAE226 was the most effective drug in causing a reduction of pTyr397FAK in HepG2, and the second one for efficacy in Huh7 cells. Based on these findings, we selected TAE226 as the best among FAKi to be studied alone and in combination with SOR in HCC models.

### TAE226 in combination with SOR efficiently reduces HCC cell viability and proliferation

In order to assess the timing for evaluating TAE226 and SOR combination effects we treated HepG2 and Huh7 cells with IC50 of each inhibitor up to 5 days of treatment. A statistically significant decrease of cell viability occurred after just 2 days of treatment with both drugs, and was further enhanced after 5 days of treatment (Fig. S2).

**Fig. 2:**
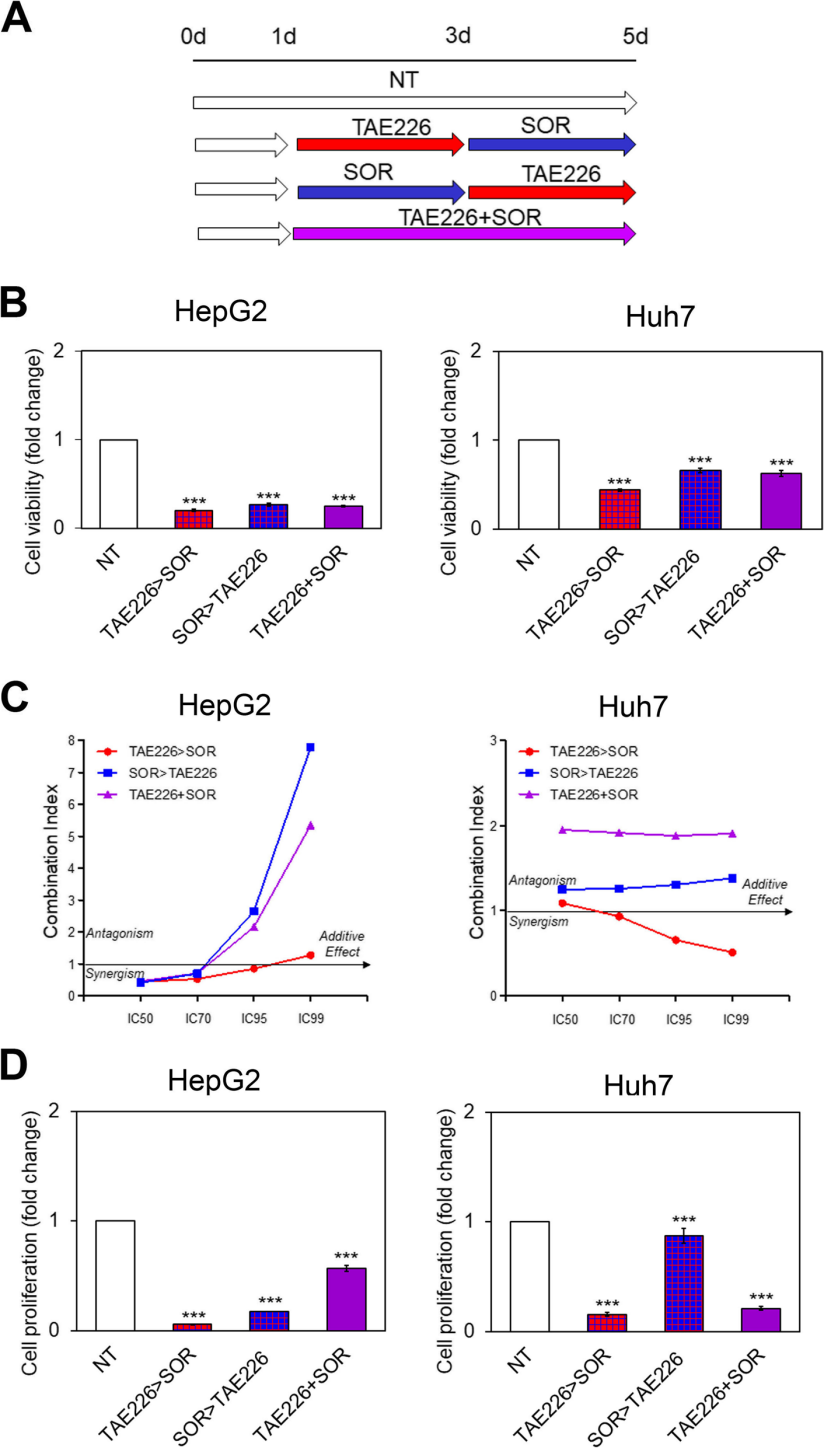
Viability and proliferation in HCC cells after treatment with different combination of TAE226 and SOR. **A** Scheme of the experimental design. **B** Cell viability was expressed as fold change of absorbance values by XTT assay, in HepG2 and Huh7 cells treated vs. NT. Values are the mean OD ± SD of three independent experiments repeated at least in triplicate. Data were analysed by 2-tailed Student’s t test. ***p < 0.001. **C** Combination Index (CI) was calculated by the Chou-Talalay method. Data plotted are CI at 50, 70, 95 and 99% fraction killed. **D** Cell proliferation was expressed as fold change of Europium (Eu) counts of BrdU incorporation in HepG2 and Huh7 cells treated vs. NT. Values are the mean Eu counts ± SD of three independent experiments repeated at least in triplicate. Data were analysed by 2-tailed Student’s t test. ***p < 0.001

Afterwards, to identify the combination of the two drugs (TAE226 and SOR) with major effects on cell viability, HCC cells were cultured in the medium for: i) 5 days (NT); ii) 1 day, then treated for 2 days with TAE226 alone, and finally exposed to SOR alone until day 5 (TAE226 > SOR); iii) 1 day, then treated for 2 days with SOR, and finally exposed to TAE226 alone until day 5 (SOR > TAE226); iv) 1 day, then treated for 4 days contemporaneously with TAE226 and SOR (TAE226 + SOR); as schematic represented in Fig. 2A. As shown in Fig. 2B and C, the combination therapy TAE226 > SOR was the most efficient into reducing cell viability. Also, the same combination therapy was the most effective in decreasing cell proliferation (Fig. 2D) compared to the other combinations and to NT cells.

### TAE226 plus SOR combination treatment impairs FAK phosphorylation at Tyr397 and enhances in vitro antitumor effect

**Fig. 3:**
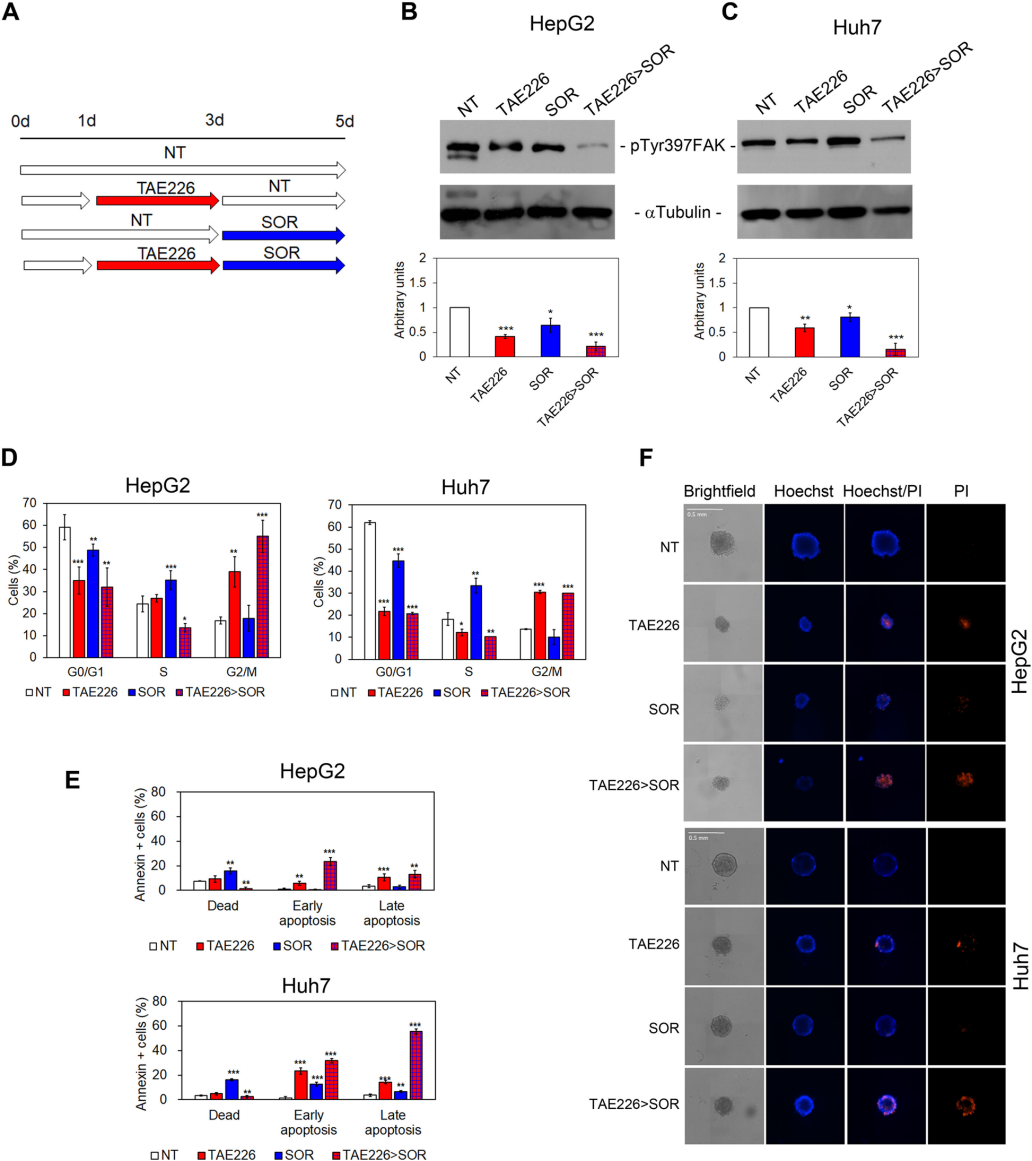
Evaluation of pTyr397FAK, cell cycle, apoptosis and TS morphology in HCC cells after TAE226 > SOR treatment. **A** Scheme of the experimental design. Representative immunoblot and quantitative analysis of pTyr397FAK expression after 48 h from treatment with the different drugs, in HepG2 (**B**) and Huh7 cells (**C**). αTubulin served as loading control. Values are the mean arbitrary units ± SD of at least three independent experiments. Data were analysed by 2-tailed Student’s t test. *p < 0.05; **p < 0.01; ***p < 0.001 vs. NT. **D** Percentage of HCC cells in G0/G1, S and G2/M phase of the cell cycle by PI staining and flow cytometry analysis. Data are expressed as mean ± SD of at least three independent experiments and was analysed by 2-tailed Student’s t test. *p < 0.05; **p < 0.01; ***p < 0.001 vs. NT. **E** Percentage of HCC cells dead or in early and late apoptosis measured by Annexin V staining and flow cytometry. Data are expressed as mean ± SD of at least three independent experiments and was analysed by 2-tailed Student’s t test. **p < 0.01; ***p < 0.001 vs. NT. **F** Representative brightfield and fluorescent images (Hoechst and PI) of multicellular TS from HepG2 and Huh7 cells NT and after treatments

Next, we evaluated the downstream effects of the selected drug-combination (TAE226 > SOR) compared with those of TAE226 and SOR alone and with NT cells (Fig. 3A).

Immunoelectrophoretic profile and densitometric analysis revealed that HCC cells treated with combination therapy TAE226 > SOR exhibited a strong reduction of pTyr397FAK protein levels (Fig. 3B and C). Moreover, as previously reported by Fukami et al. [24], TAE226 alone or in combination with SOR down-regulated the pTyr1135 form of the insulin growth factor 1 receptor β (pTyr1135IGF-1R), a well-known tyrosine kinase receptor crucial for tumour transformation and malignant cell survival (Fig. S3A and S3B).

In addition, our results demonstrated that the treatment with TAE226 as single agent or in combination with SOR (TAE226 > SOR) caused a significant reduction in the percentage of HCC cells in G0/G1 and S phase of the cell cycle, and an accumulation in G2/M phase compared to NT cells (Fig. 3D). On the contrary, SOR treatment as single agent increased the percentage of HCC cells in S phase to the detriment of those in G0/G1 phase (Fig. 3D). Data on cell death highlighted that TAE226 alone, and even more in combination with SOR (TAE226 > SOR), increased the percentage of cells in early and late apoptosis, while SOR caused cell death (Fig. 3E).

To better mimic the physiological response to treatments, their inhibitory effects were evaluated on 3D multicellular TS models of HepG2 and Huh7 cells following 48 h of treatments. Analysis of phase contrast images (brightfield) (Fig. 3F, Fig. S3C and S3D) showed that TAE226 and SOR treatments, as a single agent or in combination, caused a reduction of TS area and diameter. Moreover, TAE226 induced an increase in PI staining, indicative of TS cell death, which was further enhanced when it was in combination with SOR (Fig. 3F). Also, the antitumor effects were confirmed in colony formation assays following 28 days of treatments (Fig. S3E).

### TAE226 enhances the antitumor effect of SOR in an in vivo HCC model

**Fig. 4:**
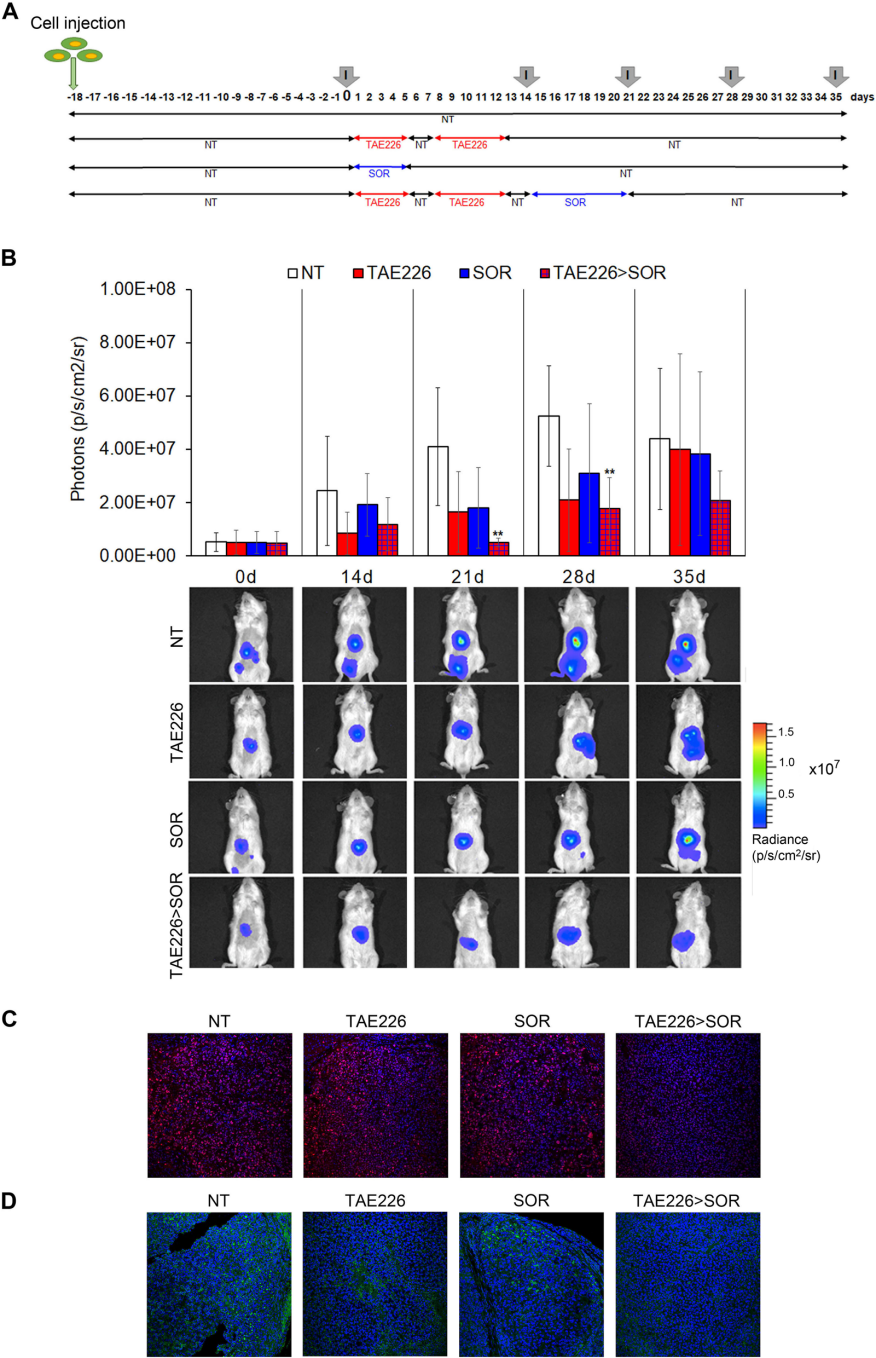
Effect of TAE226 > SOR on HCC growth in mouse xenograft model. **A** Scheme of the experimental design. Imaging analysis was performed at different times (I). **B** Quantitative analysis and representative pictures of in vivo bioluminescence imaging analysed before administration of compounds (day 0) and during treatments at days 14, 21, 28 or 35. Luminescent signals are expressed as mean ± SD of total flux of photons/sec/cm^2^/steradian (p/s/cm^2^/sr). Data were analysed by ANOVA test. (**p < 0.01; n = 6). Representative images of immunofluorescence for PCNA (in red) (**C**) and pTyr397FAK (in green) (**D**) in mouse xenograft models after treatments. The nuclei are revealed by specific DAPI staining, displayed in blue. 40X Magnification

Based on our in vitro data, we performed experiments to assess the antitumor efficacy of TAE226 > SOR combination in vivo. First, we evaluated the safe/effective dosage of TAE226 on intramuscular tumour bearing mice treated with 25 mg/kg and 50 mg/kg of TAE226, starting from day 19 after the injection of HepG2-Luc cells. The effect of the selected doses on the reduction of tumour growth was almost the same after 12 days of treatment without toxic effects (Fig. S4A), as shown by the similar levels of alanine aminotransferase (ALT) in treated mice compared to those of untreated animals (Fig. S4B). Therefore, we selected 25 mg/kg as TAE226 concentration to perform experiments on orthotopic HepG2 HCC model, and treated mice following the scheduling reported in Fig. 4A to evaluate the in vivo therapeutic efficacy of TAE226 and SOR. The antitumor efficacy was quantified using the IVIS imaging system. When TAE226 and SOR were used as single agents a transient antitumor effect was observed. A first decreased in tumour growth of about 50% was seen at 21–28 days after initiation of treatment, followed by a tumour regrowth (Fig. 4B). However, in TAE226 > SOR combination treatment a prolonged and sustained antitumor effect was observed. The tumour weight reduction was of about 90 to 70% from day 21 up to 28 (Fig. 4B). Interestingly, the efficacy of TAE226 in combination with SOR in this advanced model of human HCC was confirmed when we applied the response evaluation criteria in solid tumours [25]. In fact, stabilization of the disease for about three weeks was observed in 4/6 mice and a partial response in 1/6 mice treated with the combination (Table S2). Accordingly, as shown by immunofluorescence analysis performed on tumours at day 35 (Fig. 4C), the expression levels of proliferating cell nuclear antigen (PCNA) strongly decreased only in combination group compared to single treatments and untreated animals. Finally, as expected, the levels of pTyr397FAK expression tended to be reduced both by TAE226 and SOR alone, but were more strongly down-regulated in TAE226 > SOR (Fig. 4D).

### Combination therapy TAE226 > SOR affects the expression of cancer-related genes

**Fig. 5:**
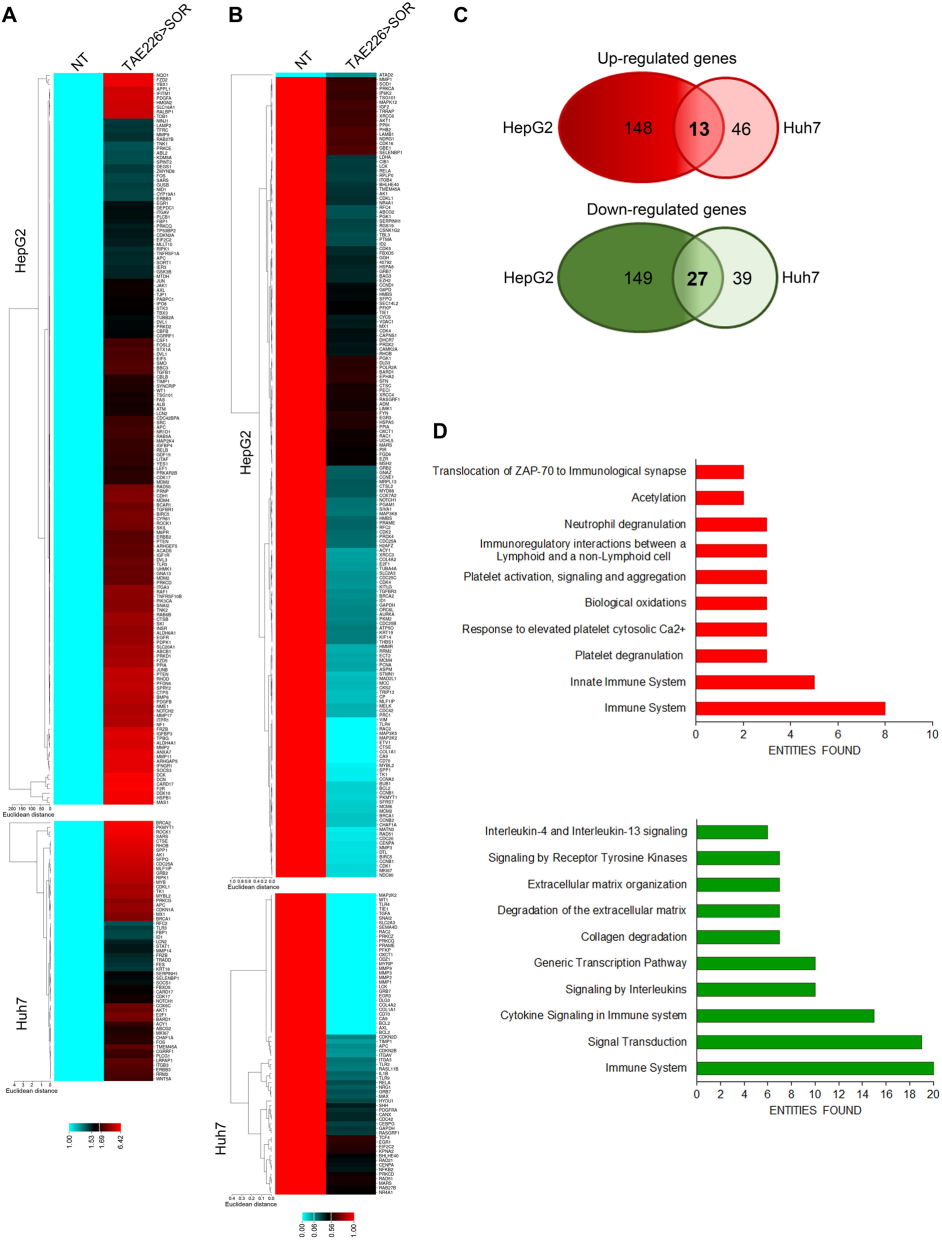
Cancer associated genes upon FAK inhibition. **A** Heatmap representation of the expression of up-regulated (**A**) and down-regulated (**B**) cancer-related genes in HepG2 and Huh7 cells in TAE226 > SOR compared to NT cells. **C** Venn diagrams showing the overlapping of up-regulated (*upper circles*) and down-regulated (*lower circles*) genes in HepG2 and Huh7 cells treated with TAE226 > SOR compared to NT cells. **D** Bar plots of the 10 most abundant pathways for commonly up-regulated (*upper plot*) or down-regulated (*lower plot*) genes in both HCC cells after treatment with TAE226 > SOR

Since we previously demonstrated that FAK silencing impacts on gene transcription [13], we evaluated if this effect was causally related to FAK catalytic activity. Thus, we analysed the effect of FAK pharmacological inhibition on the expression of a panel of cancer-related genes in HepG2 and Huh7 cells after TAE226 > SOR treatment compared to NT cells. We analysed the expression of 648 genes using a commercially available cancer open array by Real-Time PCR platform. TAE226 > SOR combined treatment induced an up-regulation of 161 genes in HepG2 cells and 59 genes in Huh7 cells (Fig. 5A), whilst it caused a down-regulation of 176 genes in HepG2 cells and 66 genes in Huh7 cells (Fig. 5B). Venn diagrams (Fig. 5C) showed that a set of 13 and 27 potential direct/indirect specific targets were respectively up-regulated and down-modulated in both cell lines compared to NT cells. Next, we performed Reactome enrichment analysis, considering separately the common up-regulated genes and those down-regulated (Table S3). As shown in Fig. 5D, among the enriched terms, we found up-regulated pathways related to biological oxidations and acetylation, and down-regulated pathways associated to signal transduction, gene transcription and extracellular matrix (ECM) degradation. Genes belonging to the immune system were both up- and down-regulated.

### Combination therapy TAE226 > SOR affects the tri-methylation of lysine 27 on histone H3 and nuclear amount of pTyr397FAK

**Fig. 6:**
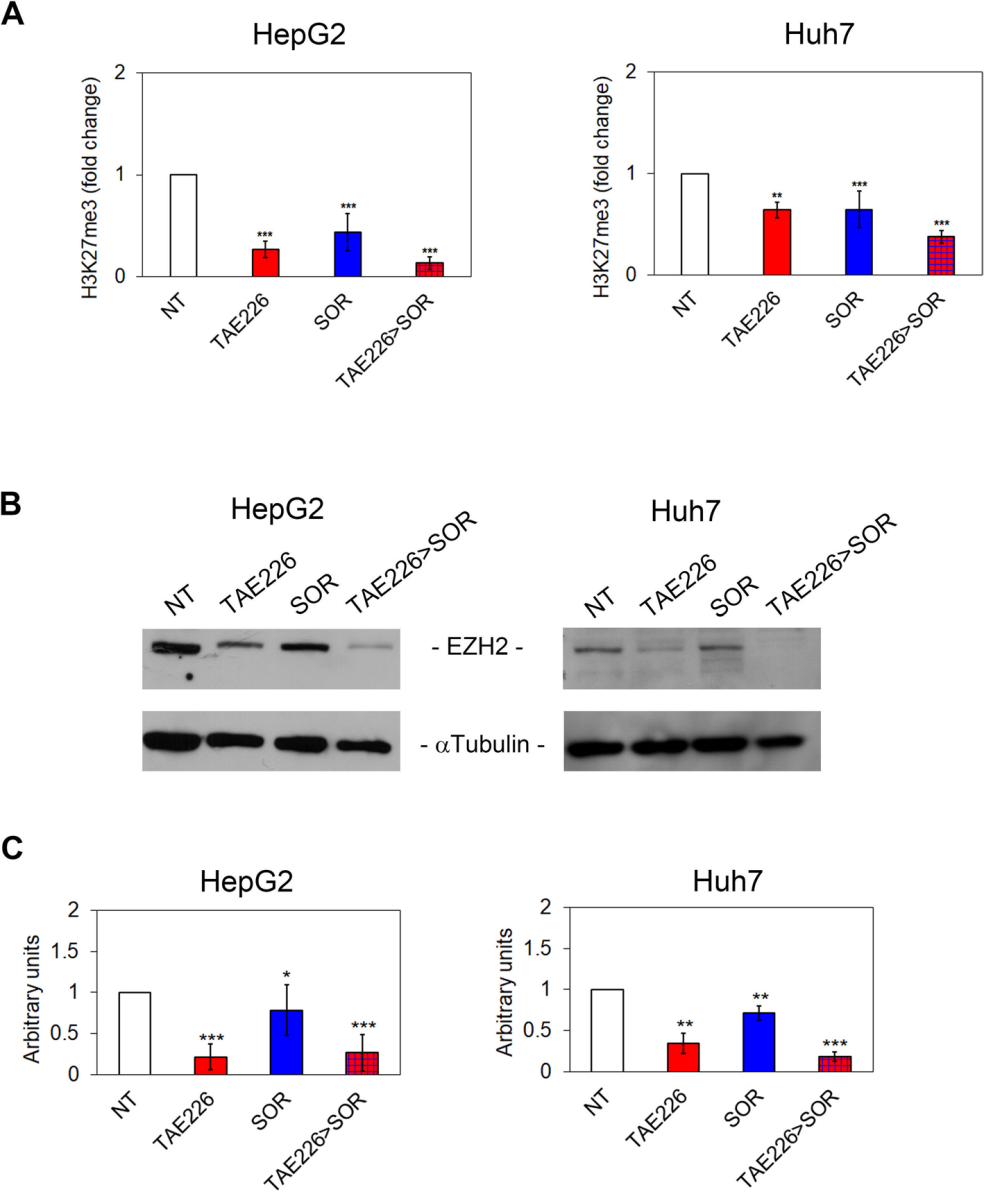
Effect of TAE226 > SOR on H3K27me3. **A** H3K27me3 levels measured by AlphaLISA assay and represented as fold change in HepG2 and Huh7 cells treated vs. NT. Data were analysed by 2-tailed Student’s t test. **p < 0.01; ***p < 0.001 vs. NT. Representative immunoblot (**B**) and quantitative analysis (**C**) of EZH2 expression after treatments, in HepG2 and Huh7 cells. αTubulin served as loading control. Values are the mean of arbitrary units ± SD of at least three independent experiments. Data were analysed by 2-tailed Student’s t test. *p < 0.05; **p < 0.01; ***p < 0.001 vs. NT

We previously observed that FAK-dependent deregulation of gene expression results in a reduced *EZH2* expression and tri-methylation of lysine 27 on histone H3, with consequent effects on gene transcription [13]. Therefore, we explored the effect of TAE226 inhibitor alone or in combination with SOR on the same pathway. As shown in Fig. 6A, AlphaLISA assay demonstrated that H3K27me3 was significantly down-regulated by TAE226 or SOR treatment in both HCC cell lines. This effect was more pronounced in cells cultured with the TAE226 > SOR combination treatment. Accordingly, EZH2 protein expression levels were significantly decreased by all treatments (Fig. 6B and C). These findings support the hypothesis that pTyr397FAK is deeply involved in the observed effects on H3K27me3. However, we still needed to explore cellular compartments in which the combination treatment plays its antitumor effects in HCC cells.

**Fig. 7:**
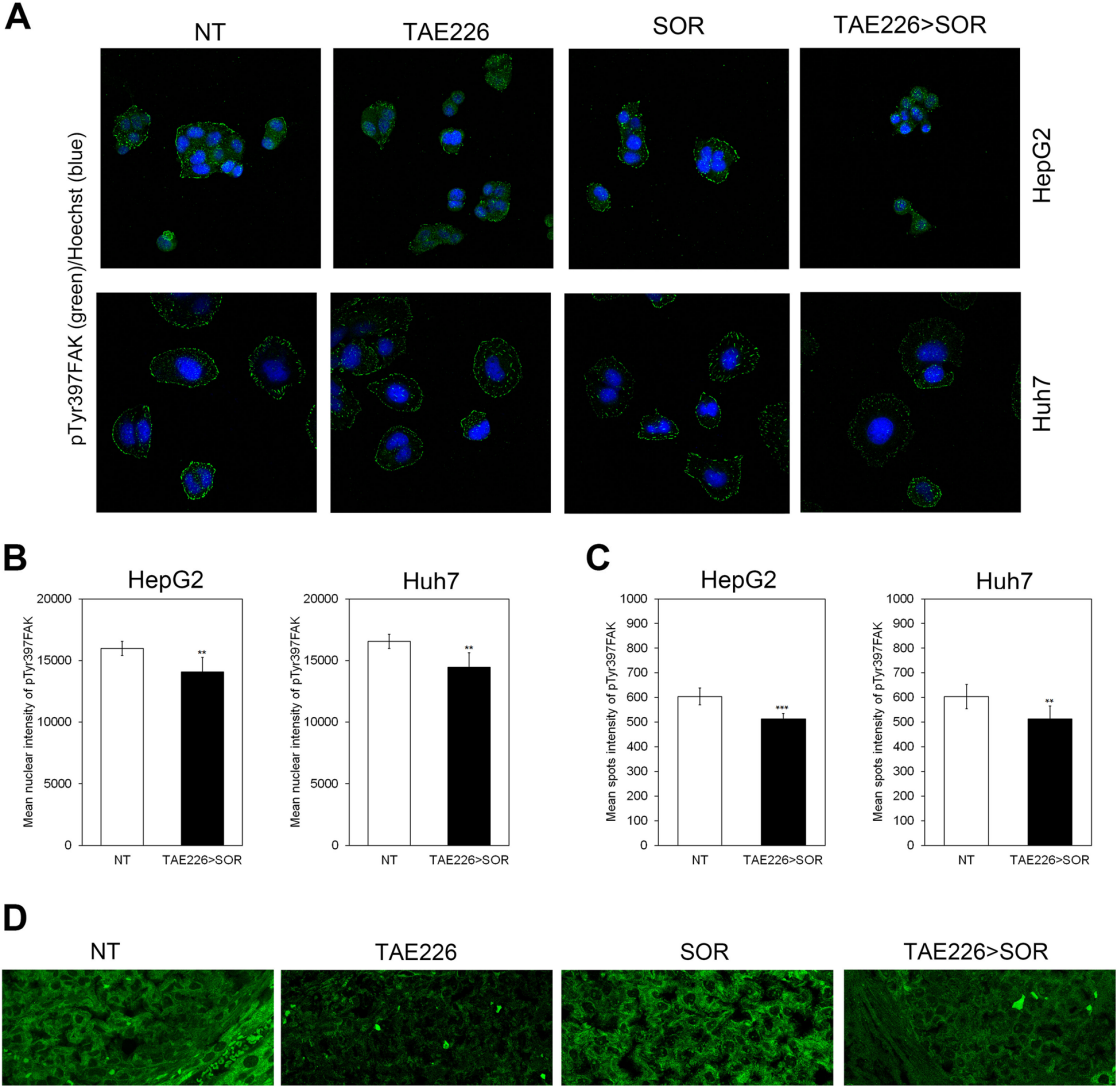
Effect of TAE226 > SOR on nuclear amount of pTyr397FAK. **A** Representative images of immunofluorescence for pTyr397FAK (in green) into the nuclei and at focal adhesions after treatments, in HepG2 and Huh7 cells. The nuclei are revealed by specific Hoechst staining (in blue). 40X Magnification. Content of pTyr397FAK into the nuclei (**B**) and at focal adhesions (**C**) in HepG2 and Huh7 cells, quantified by using the Operetta CLS in confocal mode. 40X Magnification. Values are the mean of arbitrary units ± SD of at least three independent experiments. Data were analysed by 2-tailed Student’s t test. *p < 0.05; **p < 0.01; ***p < 0.001 vs. NT. **D** Representative images of immunofluorescence for pTyr397FAK (in green) in xenograft models of HCC. 60X Magnification

Representative images showed that TAE226 > SOR reduced nuclear and focal adhesion localization of pTyr397FAK in HCC cell lines (Fig. 7A). The content of pTyr397FAK into the nuclei and at focal adhesions was quantified using Operetta CLS, an automated imaging system (Fig. 7B and C). Similarly, in the xenograft model of HCC, the treatment with TAE226 > SOR caused a down-regulation of pTyr397FAK levels in the cytoplasm, at the focal contacts, and nuclei (Fig. 7D).

### Combination therapy TAE226 > SOR reduces pTyr397FAK nuclear interaction with specific epigenetic regulators

**Fig. 8:**
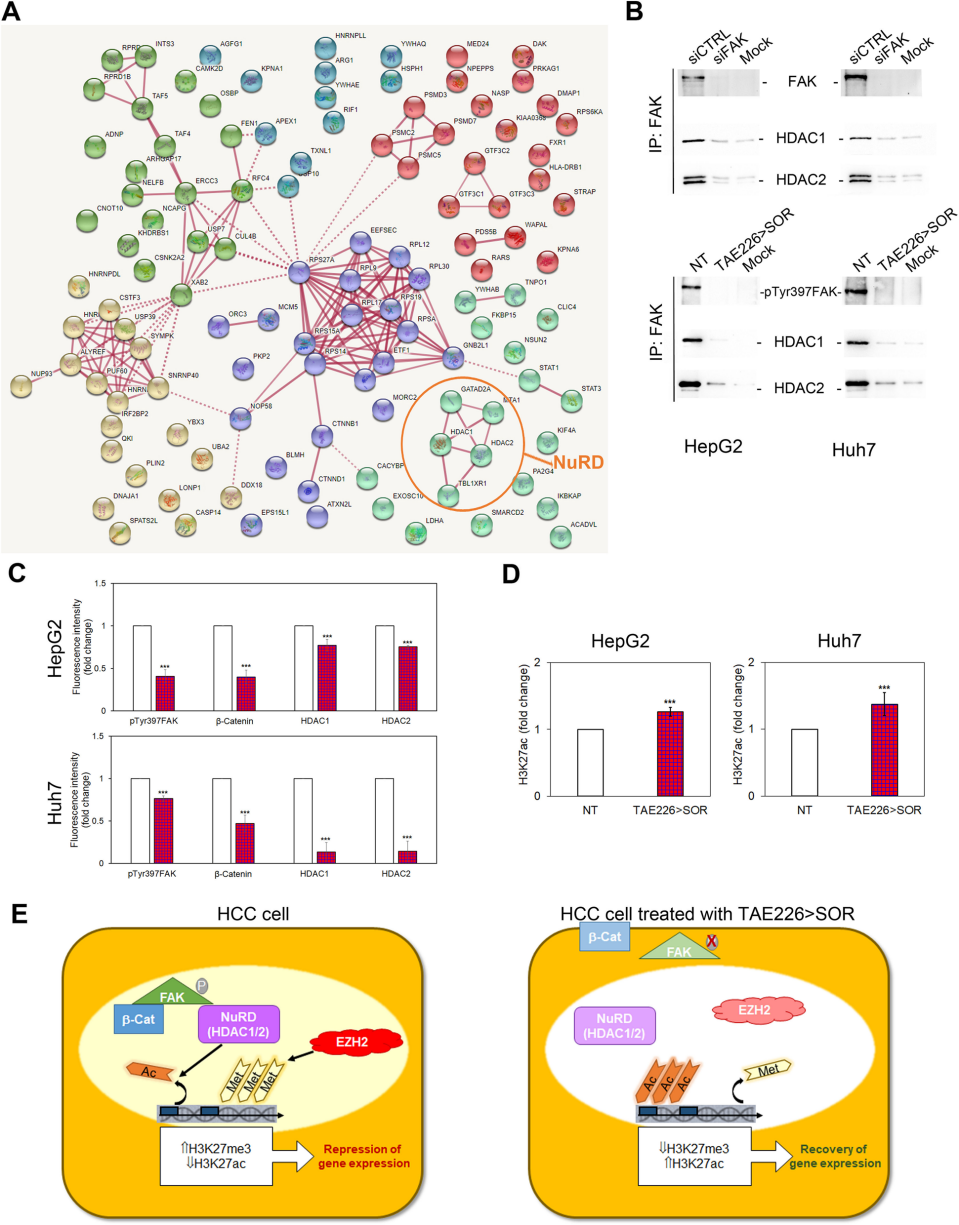
Effects of FAK inhibition on its nuclear interactome. **A** PPI networks of the nuclear FAK interactors. **B** Representative immunoblot of nuclear extracts immunoprecipitated with antibody against FAK and then immunoblotted with FAK, HDAC1, and HDAC2 antibodies in HCC cells silenced or not for FAK and in MOCK (*upper panels*); representative immunoblot of nuclear extracts immunoprecipitated with antibody against FAK and then immunoblotted with pTyr397FAK, HDAC1 and HDAC2 antibodies in NT or TAE226 > SOR HCC cells, and in MOCK (*lower panels*). The experiments were repeated in duplicate (**C**) Quantitative imaging of immunofluorescence for pTyr397FAK, β-Catenin, HDAC1 and HDAC2 represented as fold change of mean fluorescence intensity in HepG2 and Huh7 cells treated vs. NT. Data were analysed by 2-tailed Student’s t test. ***p < 0.001 vs. NT. **D** H3K27ac levels measured by AlphaLISA assay and represented as fold change in HepG2 and Huh7 cells treated vs. NT. **E** Graphical abstract reporting the interaction of FAK with NuRD complex

We previously suggested that the effect of FAK on H3K27me3 could be mediated by direct and/or indirect functional interactions between FAK and EZH2 [13]. However, the mechanism by which pTyr397FAK may influence H3 tri-methylation remained to be explored. In order to identify the nuclear interactors of FAK that could participate to the epigenetic control of gene expression, we performed proteomics on nuclear immunoprecipitates of FAK obtained from HepG2 and Huh7 cells (Fig. S5A), and the identified proteins were used for further analysis (Fig. S5B). We identified 116 putative nuclear binding partners of FAK (Table S4), including the well-known β-catenin functional interactor of FAK [26]. To better understand the nuclear interactome of FAK and its crosstalk with known protein and pathways, we constructed the interaction network containing nodes corresponding to FAK interacting proteins and further integrated the information on their associated pathways by using STRING database [23]. Six PPI networks were generated (Fig. 8A). GO annotations (Table S5) suggested that FAK interactors were mainly enriched in molecular functions related to protein stability (proteasome), RNA splicing (spliceosome), and DNA binding (epigenetic regulators). Among these proteins, some components of nucleosome remodelling and deacetylase (NuRD) complex, a chromatin-remodelling complex having a histone deacetylase activity, emerged as new and attractive nuclear interactors of FAK. Indeed, as demonstrated by immunoprecipitation experiments, nuclear FAK interacted with the deacetylase NuRD-proteins HDAC1 and HDAC2, and FAK silencing or TAE226 > SOR abolished this interaction (Fig. 8B). Moreover, in HCC cells treated with TAE226 > SOR the decrease of pTyr397FAK levels was associated with a reduction of β-catenin, HDAC1 and HDAC2 (Fig. 8C and Fig. S6). The HDACs nuclear impairment after TAE226 > SOR treatment in both HCC cells was coupled with the consequent up-regulation of H3K27ac (Fig. 8D).

## Discussion

In this study, we provided a clear and comprehensive preclinical evaluation of a FAK inhibitor, TAE226, studied in HCC cells in order to explore its potential use as first-line therapy in combination with SOR.

We found that TAE226 treatment alone reduced growth and increased apoptosis of 2D and 3D HCC in vitro models, and this effect was enhanced in combination with SOR. Moreover, TAE226 slowed down tumour growth of human HCC xenografts in vivo, and again this effect was enhanced in combination with SOR. No side effects were observed in single agent or in combination treatments.

FAK is a highly conserved 125 kDa non-receptor tyrosine kinase, mainly localized to cellular focal contacts, playing a master role in adhesion-dependent cell motility, survival and proliferation, in response to integrin and growth factor receptor signalling via its kinase-dependent and scaffold functions [27, 28]. During the last decade, the role of FAK in tumours and the utility of its inhibitors as potential therapeutic agents have been extensively investigated [29]. FAK was identified as an independent risk factor for HCC, with its overexpression predicting poor prognosis in HCC patients [10, 30]. However, to date there is a little evidence of the role of FAK activation, and studies on the effect of clinically translatable FAKi are still in embryo for this tumour. We previously demonstrated that FAK silencing may reduce in vitro and in vivo HCC growth by affecting the expression of cancer-promoting genes, including the pro-oncogenic EZH2 [13]. Moreover, it has been reported that FAK may play a key role in the control of liver cancer stem cells proliferation, and its inhibition and functional interaction with β-Catenin has recently been identified as a potential strategy to overcome SOR-related resistance in this cell subpopulation [30–32]. All these findings suggest that the network between FAK, epigenetic modifications and β-Catenin might be a useful therapeutic target to treat HCC resistant to SOR.

Here, we demonstrated that TAE226 strongly reduced HCC growth and that in combination with SOR it enhanced its antitumor efficacy overcoming the mechanisms of resistance to SOR. The higher efficiency of TAE226 in reducing HCC growth respect to other FAKi could be linked to its ability of targeting also IGF-1R phosphorylation and activation [24].

We found that combination therapy TAE226 plus SOR reduced HCC growth both in vitro and in vivo and affected the expression of tumour-promoting genes and multifunctional epigenetic changes via de-regulation of the nuclear interactome of FAK. In particular, combination therapy induced changes in genes involved in the immune system, signal transduction, ECM degradation, and intriguingly emerged the effect on acetylation regulators. Interestingly, the effect of TAE226 > SOR on ECM degradation may corroborate the possible use of FAKi as an effective approach in patients with specific patterns, such as patients with high expression of COL4A1 [33].

The epigenetic effect of FAK depletion in HCC anticipated by our previous study has been confirmed by the strong reduction of EZH2 protein expression and H3K27me3 after TAE226 plus SOR combined treatment [13].

The multiple epigenetic effects of FAK inhibition suggested that this protein might exert some of these functions in the nuclear compartment. Accordingly, several lines of evidence have demonstrated that, into the nucleus, activated FAK may control different transcription factors leading to changes in gene expression [34]. Our work demonstrated that the nuclear localization of the Tyr397 phosphorylated form of FAK in HCC cells was reverted by the combined treatment TAE226 plus SOR in both in vitro and in vivo models. Moreover, to define the nuclear interactome of FAK in HCC, we performed a proteomic analysis. The results of proteomic study confirmed the evidence of multiple functional but also physical interactions of FAK with β-catenin, spliceosome, proteasome, and epigenetic regulators [13, 35–37]. Noteworthy, in our study the physical interaction of FAK with the NuRD complex emerged as a great novelty of the nuclear interactome of FAK. The NuRD complex consists of different subunits, including histone deacetylase HDAC1/HDAC2, the histone demethylase KDM1A, MTA1/MTA2/MTA3, GATAD2A/GATAD2B, RBBP4/RBBP7, MBD2/MBD3, and the ATP-dependent chromatin remodelling helicase CHD3/CHD4 [38]. Although NuRD complex has been linked to many aspects of oncogenesis, the expression and regulation of its subunits are not completely understood in HCC [39–41].

Our results highlighted that either FAK depletion by silencing or combined treatment TAE226 plus SOR decreased the nuclear amount of HDAC1/2, reducing their activity by an increase of H3K27Ac. It is conceivable that the increased acetylation of histone H3 lysine 27 observed under the treatment with TAE226 plus SOR may act as a recovery mechanism of the expression of tumour suppressor genes that are maintained inactive by histone H3 lysine 27 methylation in HCC. However, this aspect requires further investigation. Finally, our results suggest that FAKi may represent with epigenetic drugs (i.e. HDAC inhibitors) a promising co-targeting opportunity in HCC [42].

## Conclusions

In conclusion, as reported in Fig. 8E, we found that TAE226 inhibitor of FAK combined with SOR slows down HCC growth by multifunctional epigenetic effects, which mainly include the reduction of FAK nuclear levels and its detrimental activity on histone H3 acetylation. Taken together, our findings provide the evidence that the modulation of FAK nuclear interactome may lead to new promising therapeutics for HCC [36].

## Supplementary Information

**Additional file 1: Table S1**. List of antibodies.

**Additional file 2: Table S2**. In vivo antitumor efficacy of TAE226>SOR combination on orthotopic liver tumour.

**Additional file 3: Table S3**. Common up- and down-regulated genes.

**Additional file 4: Table S4**. Nuclear interactors of FAK.

**Additional file 5: Table S5**. GO annotations.

**Additional file 6: Figure S1**. FAK phosphorylation in HCC cells after IC50 values of treatments. Quantitative analysis of pTyr397FAK expression after 48 h of treatment with the different drugs, in HepG2 and Huh7 cells. Values are the mean arbitrary units ± SD of at least three independent experiments. Data were analysed by 2-tailed Student’s t test. *p < 0.05; ***p < 0.001 vs. NT cells.

**Additional file 7: Figure S2**. Evaluation of the IC50 values of TAE226 and SOR on HCC cell viability after 2 and 5 days. Cell viability, measured by XTT assay, after 2 and 5 days from treatment with TAE226 or SOR, in HepG2 and Huh7 cells. Values are the mean OD ± SD of three independent experiments repeated at least in duplicate. Data were analysed by ANOVA. ***p < 0.001.

**Additional file 8: Figure S3**. IGF-1R in HCC cells after treatment with TAE226 > SOR compared to single treatments. Representative immunoblot and quantitative analysis of phosphorylated IGF-1R after 48 h from treatment with the different drugs, in HepG2 (A) and Huh7 cells (B). αTubulin served as loading control. Values are the mean arbitrary units ± SD of at least three independent experiments. Data were analysed by 2-tailed Student’s t test. **p < 0.01; ***p < 0.001 vs. NT. Endpoints graphs representing average area (C), and diameter (D) of TS after 48 h of treatment expressed as fold induction. Data are expressed as mean ± SD of two independent experiments repeated in triplicate and were analysed by 2-tailed Student’s t test. *p < 0.05; **p < 0.01; ***p < 0.001 vs. NT. (E) Histograms of colony formation assay performed in HCC cells after treatments. Values are the mean ± SD of at least three independent experiments. Data were analysed by 2-tailed Student’s t test. *p < 0.05; ***p < 0.001 vs. NT.

**Additional file 9: Figure S4**. Pilot study for quantitative setting of TAE226 concentration in a heterotopic HepG2 xenograft model of HCC. (A) Quantitative analysis of tumour weight at various time points in NT animals or after TAE226 treatments. *P* values were calculated at day 12 using an unpaired two-tailed t-test, between treated and untreated tumours. **** *p*<0.0001. (B) Quantitative analysis of serum liver enzyme alanine aminotransferase (ALT) levels, expressed as U/L, in NT animals or after TAE226 treatments.

**Additional file 10: Figure S5**. Analysis of FAK nuclear interaction with specific epigenetics regulators. (A) Coomassie-stained 1D SDS-PAGE gels used for the in-gel digestions of nuclear immunoprecipitates of FAK in HCC cells. The photographs showed gel lanes excision into 10 bands for mass spectrometry characterization of FAK nuclear interactors. (B) Pipeline employed for the analysis of proteomics data.

**Additional file 11: Figure S6**. pTyr397FAK nuclear interactors before and after treatment with TAE226 > SOR. Representative images of immunofluorescence for pTyr397FAK, β-Catenin, HDAC1 and HDAC2 in HepG2 and Huh7 cells NT and after TAE226 > SOR treatment. 60X Magnification.

## Data Availability

The datasets used and/or analysed during the current study are available from the corresponding author on reasonable request.

## Abbreviations

HCC: Hepatocellular carcinoma
SOR: Sorafenib
FAK: Focal adhesion kinase
FDA: Food and drug administration
TACE: Transarterial chemoembolization
pTyr397FAK: Phosphorylated at Tyr-397 form of FAK
EZH2: Enhancer of zeste homolog 2
FAKi: FAK inhibitors
CI: Combination index
BrdU: 5-bromo-2’deoxyuridine
DELFIA: Dissociation-enhanced lanthanide fluorescent immunoassay
PI: Propidium iodide
WB: Western blotting
TS: Tumour spheroids
RQ: Relative quantity
FDR: False discovery rate
H3K27me3: Tri-methyl-histone H3 lysine 27
RT: Room temperature
DTT: Dithiothreitol
PPI: Protein-protein interaction
GO: Gene ontology
H3K27ac: Acetylated-histone H3 lysine 27
NT: Non-treated
pTyr1135IGF-1R: Phosphorylated at Tyr1135 form of the insulin growth factor 1 receptor β
ALT: Alanine aminotransferase
PCNA: Proliferating cell nuclear antigen
ECM: Extracellular matrix
NuRD: Nucleosome remodelling and deacetylase
SD: Standard deviation
Eu: Europium
