# Correction to: Focal adhesion kinase inhibitor TAE226 combined with Sorafenib slows down hepatocellular carcinoma by multiple epigenetic effects

**DOI:** 10.1186/s13046-022-02247-y

**Published:** 2022-01-27

**Authors:** Ilaria Romito, Manuela Porru, Maria Rita Braghini, Luca Pompili, Nadia Panera, Annalisa Crudele, Daniela Gnani, Cristiano De Stefanis, Marco Scarsella, Silvia Pomella, Stefano Levi Mortera, Emmanuel de Billy, Adrian Libenzio Conti, Valeria Marzano, Lorenza Putignani, Manlio Vinciguerra, Clara Balsano, Anna Pastore, Rossella Rota, Marco Tartaglia, Carlo Leonetti, Anna Alisi

**Affiliations:** 1 Unit of Molecular Genetics of Complex Phenotypes, Bambino Gesu Children’s Hospital, IRCCS, Via S. Paolo, 15, 00146 Rome, Italy; 2 Unit of Oncogenomic and Epigenetic, IRCCS Regina Elena National Cancer Institute, Rome, Italy; 3 Experimental Imaging Center, IRCCS San Raffaele Scientific Institute, 20132 Milan, Italy; 4 Core Facilities, Bambino Gesu Children’s Hospital, IRCCS, Rome, Italy; 5 Department of Paediatric Haematology/Oncology and Cellular and Gene Therapy, Bambino Gesu Children’s Hospital, IRCCS, Rome, Italy; 6 Unit of Human Microbiome, Multimodal Laboratory Medicine Research Area, Bambino Gesu Children’s Hospital, IRCCS, Rome, Italy; 7 Unit of Microbiomics, Microbiology and Immunological Diagnostics, Department of Diagnostics and Laboratory Medicine Bambino Gesu Children’s Hospital, IRCCS, Rome, Italy; 8 International Clinical Research Center, St. Anne’s University Hospital, Brno, Czech Republic; 9 Department of Translational Stem Cell Biology, Research Institute of the Medical University of Varna, 9002 Varna, Bulgaria; 10 Department of Life, Health and Environmental Sciences MESVA, University of L’Aquila, L’Aquila, Italy; 11 Francesco Balsano Foundation, Rome, Italy; 12 Research Unit of Diagnostical and Management Innovations, Bambino Gesu Children’s Hospital, IRCCS, Rome, Italy; 13 Genetics and Rare Diseases Research Division, Bambino Gesu Children’s Hospital, IRCCS, Rome, Italy

**Correction to: J Exp Clin Cancer Res 40, 364 (2021).**

**https://doi.org/10.1186/s13046-021-02154-8**

Following publication of the original article [1], the authors identified errors Figs. 3, 4, 5 and 6 specifically:

**Fig. 3:**
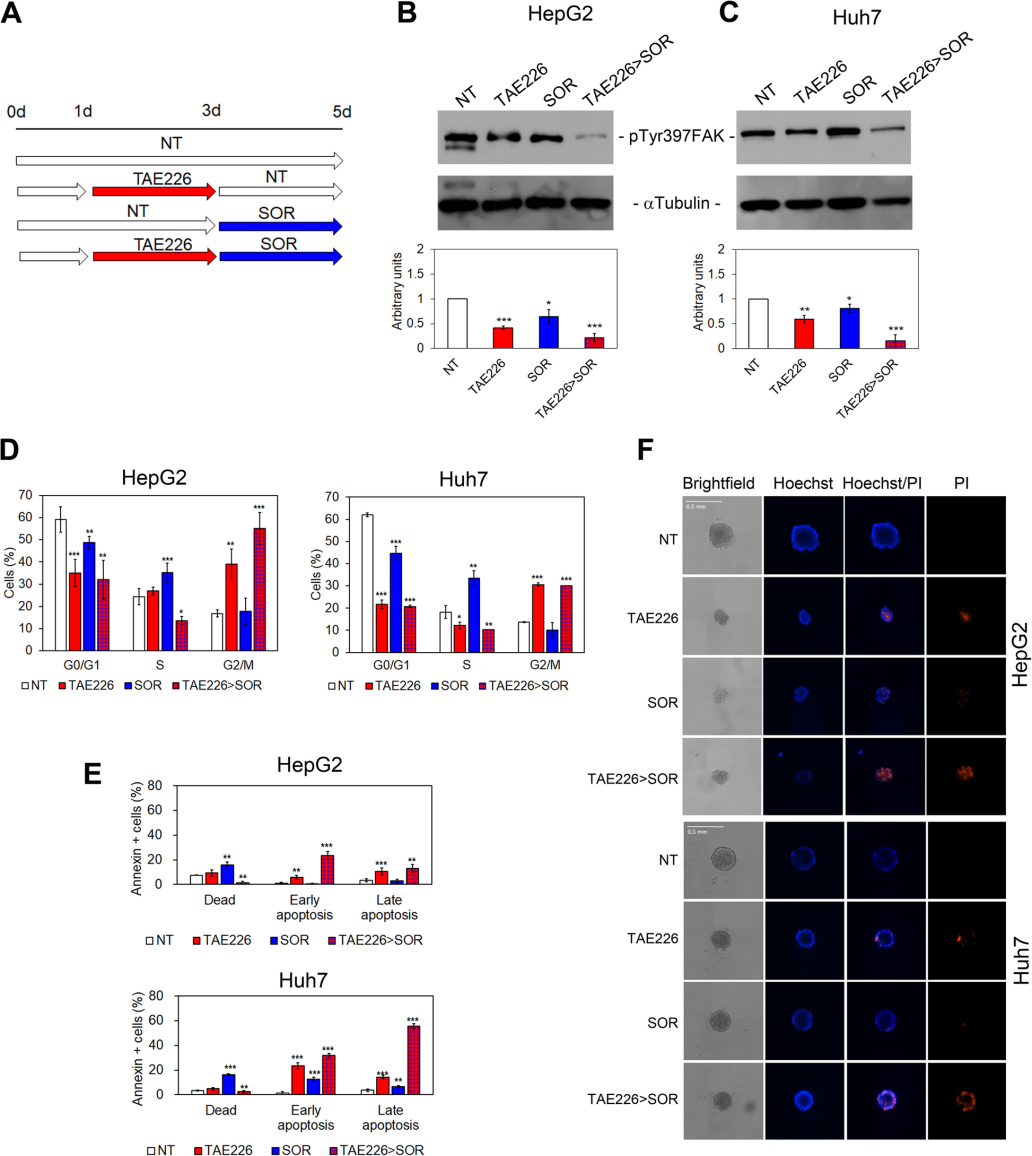
Evaluation of pTyr397FAK, cell cycle, apoptosis and TS morphology in HCC cells after TAE226 > SOR treatment. **A** Scheme of the experimental design. Representative immunoblot and quantitative analysis of pTyr397FAK expression after 48 h from treatment with the different drugs, in HepG2 (**B**) and Huh7 cells (**C**). αTubulin served as loading control. Values are the mean arbitrary units ± SD of at least three independent experiments. Data were analysed by 2-tailed Student’s t test. *p < 0.05; **p < 0.01; ***p < 0.001 vs. NT. **D** Percentage of HCC cells in G0/G1, S and G2/M phase of the cell cycle by PI staining and flow cytometry analysis. Data are expressed as mean ± SD of at least three independent experiments and analysed by 2-tailed Student’s t test. *p < 0.05; **p < 0.01; ***p < 0.001 vs. NT. **E** Percentage of HCC cells dead or in early and late apoptosis measured by Annexin V staining and flow cytometry. Data are expressed as mean ± SD of at least three independent experiments and analysed by 2-tailed Student’s t test. **p < 0.01; ***p < 0.001 vs. NT. F Representative brightfield and fluorescent images (Hoechst and PI) of multicellular TS from HepG2 and Huh7 cells NT and after treatments

**Fig. 4:**
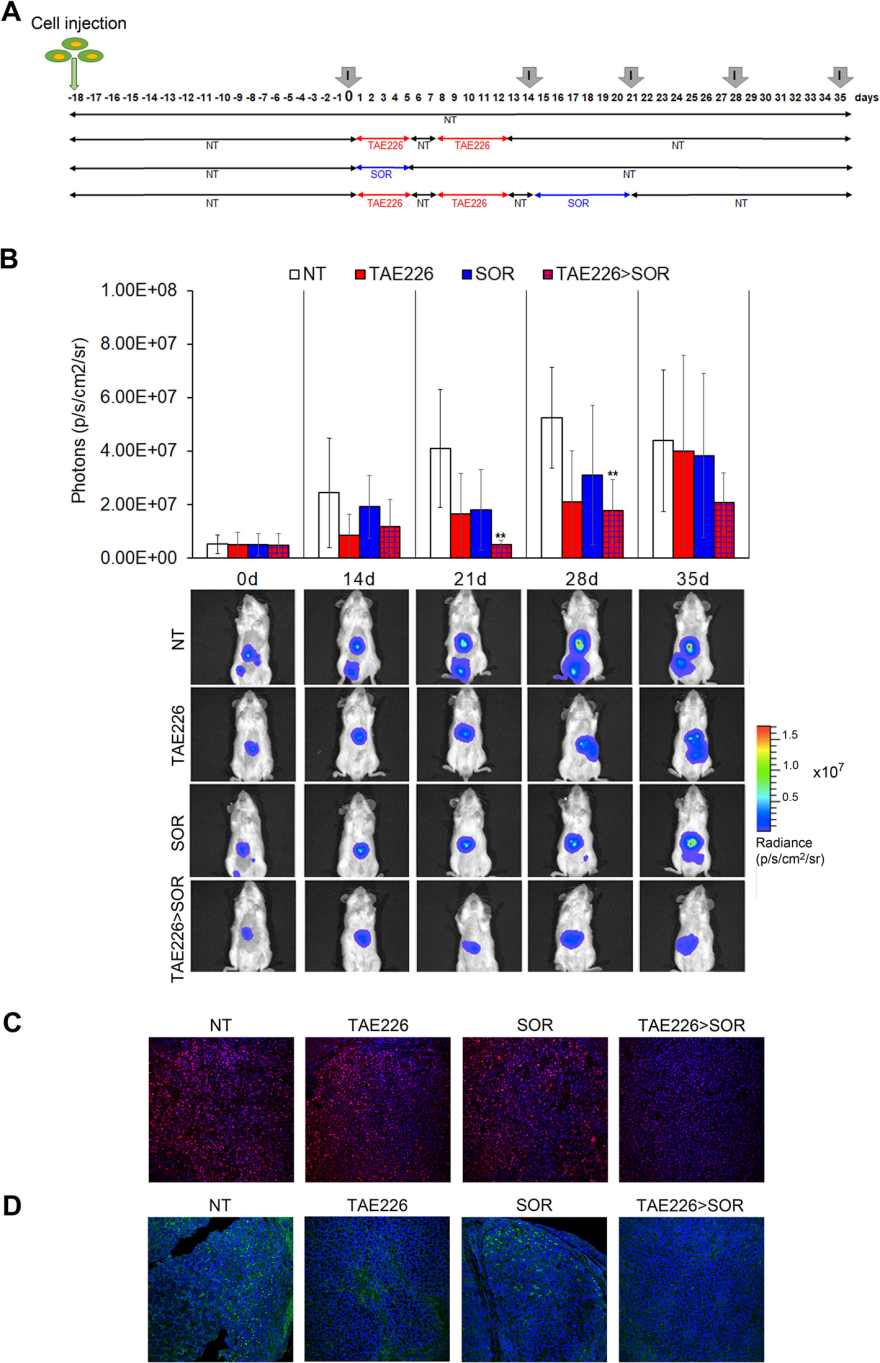
Effect of TAE226 > SOR on HCC growth in mouse xenograft model. **A** Scheme of the experimental design. Imaging analysis was performed at different times (I). **B** Quantitative analysis and representative pictures of in vivo bioluminescence imaging analysed before administration of compounds (day 0) and during treatments at days 14, 21, 28 or 35. Luminescent signals are expressed as mean ± SD of total flux of photons/sec/cm2/steradian (p/s/cm^2^/sr). Data were analysed by ANOVA test. (**p < 0.01; *n* = 6). Representative images of immunofluorescence for PCNA (in red) (**C**) and pTyr397FAK (in green) (**D**) in mouse xenograft models after treatments. The nuclei are revealed by specific DAPI staining, displayed in blue. 40X Magnification

**Fig. 5:**
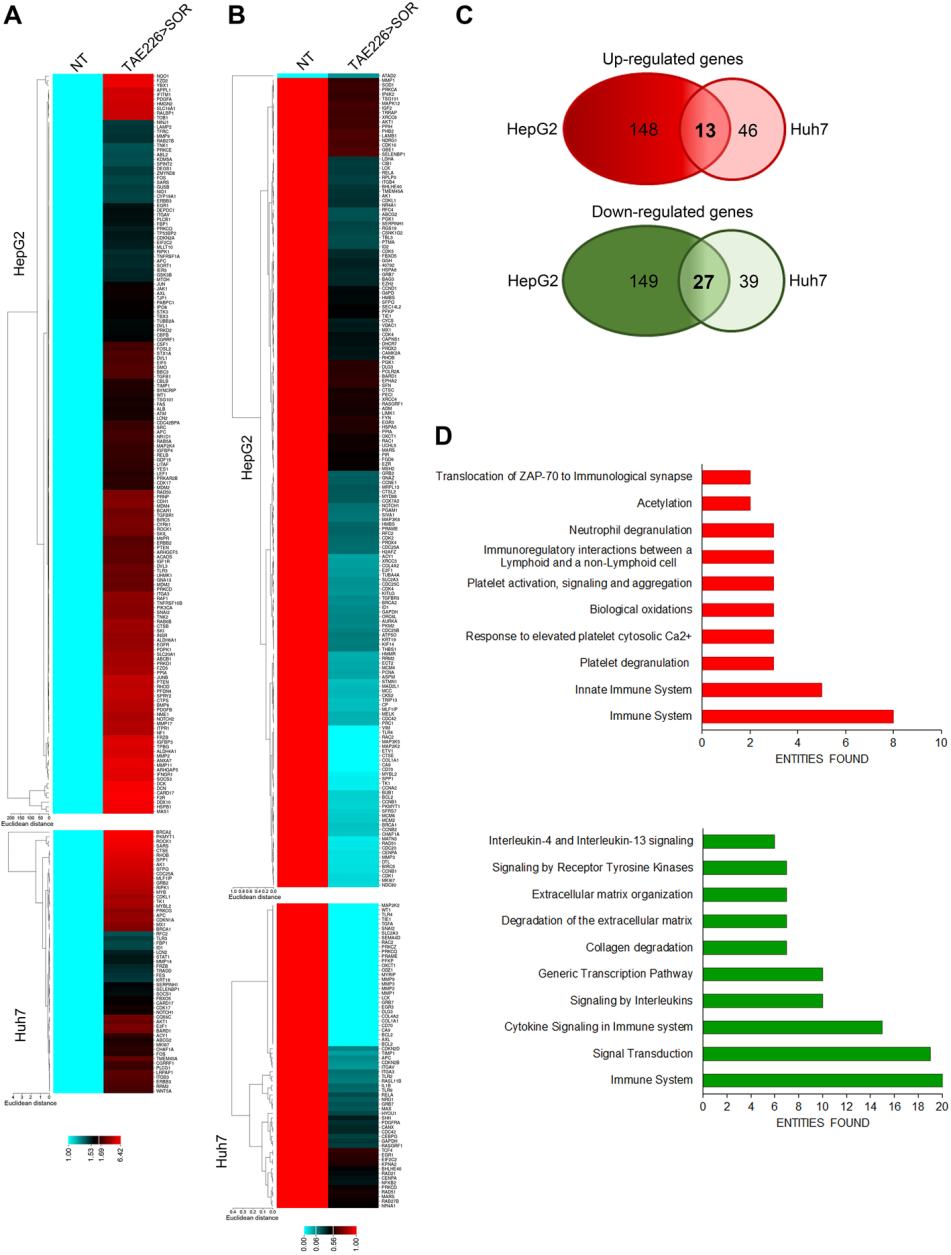
Cancer associated genes upon FAK inhibition. **A** Heatmap representation of the expression of up-regulated (**A**) and down-regulated (**B**) cancer-related genes in HepG2 and Huh7 cells in TAE226 > SOR compared to NT cells. **C** Venn diagrams showing the overlapping of up-regulated (*upper circles*) and down-regulated (*lower circles*) genes in HepG2 and Huh7 cells treated with TAE226 > SOR compared to NT cells. **D** Bar plots of the 10 most abundant pathways for commonly up-regulated (*upper plot*) or down-regulated (*lower plot*) genes in both HCC cells after treatment with TAE226 > SOR

**Fig. 6:**
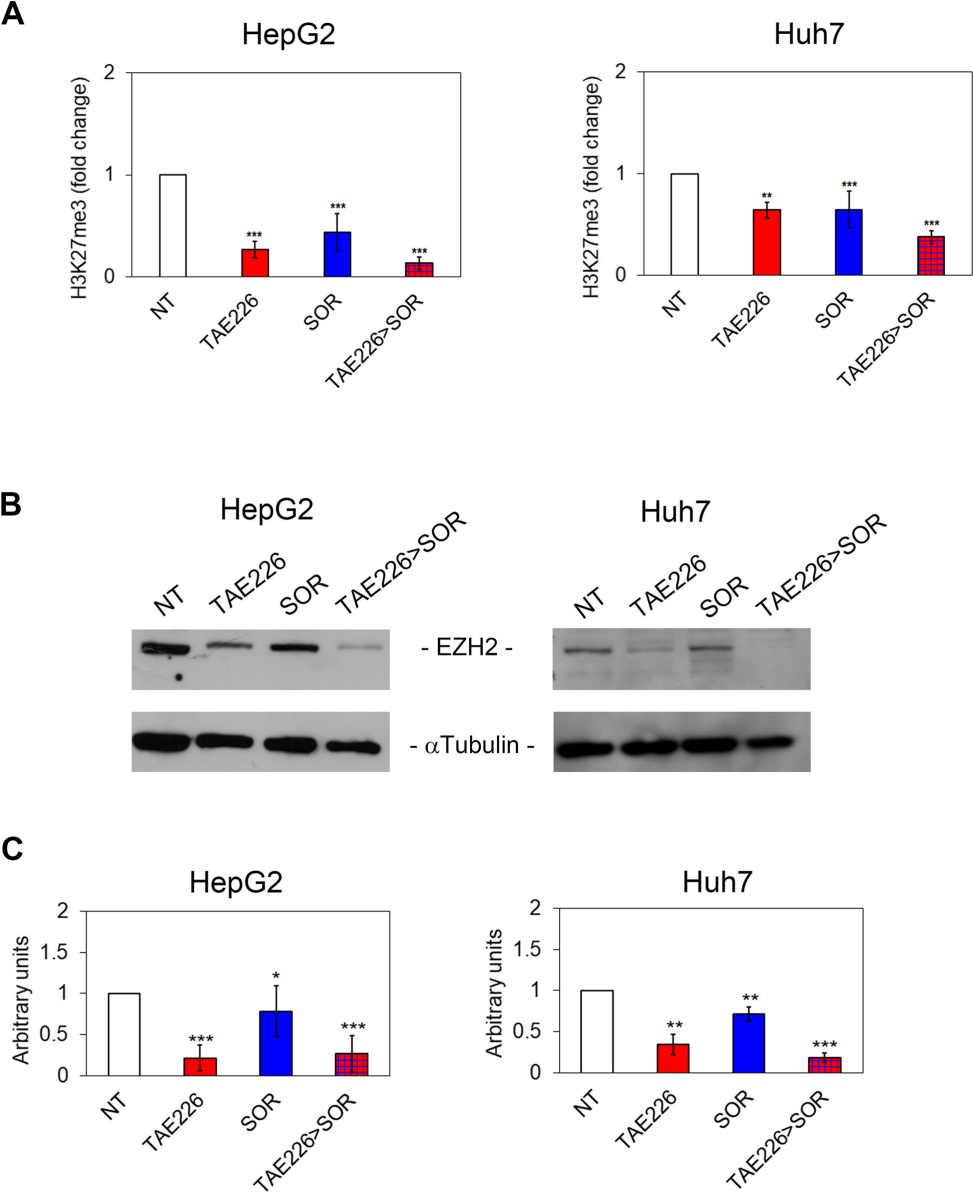
Effect of TAE226 > SOR on H3K27me3. **A** H3K27me3 levels measured by AlphaLISA assay and represented as fold change in HepG2 and Huh7 cells treated vs. NT. Data were analysed by 2-tailed Student’s t test. **p < 0.01; ***p < 0.001 vs. NT. Representative immunoblot (**B**) and quantitative analysis (**C**) of EZH2 expression after treatments, in HepG2 and Huh7 cells. αTubulin served as loading control. Values are the mean of arbitrary units ± SD of at least three independent experiments. Data were analysed by 2-tailed Student’s t test. *p < 0.05; **p < 0.01; ***p < 0.001 vs. NT

Figure 3d - Percentage of HCC cells in G0/G1, S and G2/M phase of the cell cycle by PI staining and flow cytometry analysis. Data are expressed as mean ± SD of at least three independent experiments and analysed by 2-tailed Student’s t test. *p < 0.05; **p < 0.01; ***p < 0.001 vs. NT

Figure 4b - Quantitative analysis and representative pictures of in vivo bioluminescence imaging analysed before administration of compounds (day 0) and during treatments at days 14, 21, 28 or 35. Luminescent signals are expressed as mean ± SD of total flux of photons/sec/cm2/steradian (p/s/cm2/sr). Data were analysed by ANOVA test. (**p < 0.01 *n* = 6). Representative images of immunofluorescence for PCNA (in red)

Figure 5a, b, c - Heatmap representation of the expression of up-regulated; cancer-related genes in HepG2 and Huh7 cells in TAE226 > SOR compared to NT cells; Venn diagrams showing the overlapping of up-regulated (*upper circles*) and down-regulated (*lower circles*) genes in HepG2 and Huh7 cells treated with TAE226 > SOR compared to NT cells.

Figure 6c - EZH2 expression after treatments, in HepG2 and Huh7 cells. αTubulin served as loading control. Values are the mean of arbitrary units ± SD of at least three independent experiments. Data were analysed by 2-tailed Student’s t test. *p < 0.05; **p < 0.01; ***p < 0.001 vs. NT

In addition, the revised Supplementary materials also need to be corrected specifically, Additional files 6, 7 and 8.

The authors provided the Journal with the original data files. The corrected figures are given here. The corrections do not have any effect on the final conclusions of the paper. The original article has been corrected.

## Supplementary Information

**Additional file 6.**

**Additional file 7.**

**Additional file 8.**
