# Supplementary material for: Focal adhesion kinase inhibitor TAE226 combined with Sorafenib slows down hepatocellular carcinoma by multiple epigenetic effects
Source: J Exp Clin Cancer Res. 2021 Nov 16;40:364. doi: 10.1186/s13046-021-02154-8 (PMC8597092; doi:10.1186/s13046-021-02154-8)
Supplement: Supplementary file 1 — Additional file 1: Table S1. List of antibodies. [file 13046_2021_2154_MOESM1_ESM.docx]

**Table S1. List of antibodies.**

| Target | Producer | Catalog number | Dilution |
| --- | --- | --- | --- |
| FAK | Cell Signaling Technology (Danvers, Massachusetts, USA) | 13009 | Western Blotting: 1:1000 in 2.5% w/v Nonfat Dry Milk, 1X TBS, 0.1% Tween-20.  Immunofluorescence: 1:100 in PBS/BSA 1%. |
| Phospho-FAK (Tyr397) | Cell Signaling Technology | 8556 | Western Blotting: 1:1000 in 5% w/v BSA, 1X TBS, 0.1% Tween-20. |
| Phospho-FAK (Tyr397) | Thermo Fisher Scientific Inc. (Waltham, Massachusetts, USA) | 700255 | Immunofluorescence: 1:100 in PBS/BSA 1%. |
| pTyr1135IGF-I Receptor β | Cell Signaling Technology | 3918 | Western Blotting: 1:1000 in 5% w/v BSA, 1X TBS, 0.1% Tween-20. |
| Ezh2 | BD Transduction Laboratories (Franklin Lakes, New Jersey, USA) | 612666 | Western Blotting: 1:1000 in 2.5% w/v Nonfat Dry Milk, 1X TBS, 0.1% Tween-20.  Immunofluorescence:  1:100 in PBS/BSA 1%. |
| α-Tubulin | Novus biologicals (Centennial, Colorado, USA) | NB100-690 | Western Blotting: 1:5000 in 2.5% w/v Nonfat Dry Milk, 1X TBS, 0.1% Tween-20. |
| PCNA | Cell Signaling Technology | 13110 | Immunofluorescence: 1:300 in PBS/BSA 5%. |
| HDAC1 | Cell Signaling Technology | 34589 | Western Blotting: 1:1000 in 2.5% w/v Nonfat Dry Milk, 1X TBS, 0.1% Tween-20.  Immunofluorescence:  1:100 in PBS/BSA 1%. |
| HDAC2 | Cell Signaling Technology | 5113 | Western Blotting: 1:1000 in 2.5% w/v Nonfat Dry Milk, 1X TBS, 0.1% Tween-20.  Immunofluorescence:  1:100 in PBS/BSA 1%. |
| β-Catenin | Novocastra, Leica Biosystems (Wetzlar, Germany) | NCL-L-B-CAT | Immunofluorescence:  1:100 in PBS/BSA 1%. |
| Peroxidase-AffiniPure Goat Anti-Rabbit IgG (H+L) | Jackson ImmunoResearch (Ely, Cambridgeshire, UK) | 111-035-144 | Western Blotting: 1:7000 in 2.5% w/v Nonfat Dry Milk, 1X TBS, 0.1% Tween-20. |
| Peroxidase-AffiniPure Goat Anti-Mouse IgG (H+L) | Jackson ImmunoResearch | 115-035-146 | Western Blotting: 1:7000 in 2.5% w/v Nonfat Dry Milk, 1X TBS, 0.1% Tween-20. |
| Alexa Fluor 488 conjugate, F(ab’)2-Goat anti-rabbit IgG(H+L) Secondary Antibody | Thermo Fisher Scientific Inc. | A-11070 | Immunofluorescence:  1:500 in PBS/BSA 1%. |
| Alexa Fluor 555 conjugate, F(ab’)2-Goat anti-Mouse IgG(H+L) Secondary Antibody | Thermo Fisher Scientific Inc. | A-21425 | Immunofluorescence:  1:300 in PBS/BSA 1%. |
