# Supplementary material for: Focal adhesion kinase inhibitor TAE226 combined with Sorafenib slows down hepatocellular carcinoma by multiple epigenetic effects
Source: J Exp Clin Cancer Res. 2021 Nov 16;40:364. doi: 10.1186/s13046-021-02154-8 (PMC8597092; doi:10.1186/s13046-021-02154-8)
Supplement: Supplementary file 2 — Additional file 2: Table S2. In vivo antitumor efficacy of TAE226>SOR combination on orthotopic liver tumour. [file 13046_2021_2154_MOESM2_ESM.docx]

**Table S2. *In vivo* antitumor efficacy of TAE-226>SOR combination on orthotopic liver tumour.**

| Treatment groups* | Tumour volume inhibition^&^ | Progression | Stable disease^#^ | Partial response^$^ | Body weight loss^#^ |
| --- | --- | --- | --- | --- | --- |
| TAE226 | 49 | 5/6 | 1/6 | 0/6 | 0 |
| SOR | 50 | 5/6 | 1/6 | 0/6 | 0 |
| TAE226>SOR | 89 | 1/6 | 4/6 | 1/6 | 0 |

*The mice were treated with TAE226 (*per os* at 25mg/Kg) for five consecutive days repeated for 2 weeks, SOR (*per os* at 30 mg/Kg) for five consecutive days or TAE (*per os* at 25mg/Kg) for five consecutive days repeated for 2 weeks followed by SOR (*per os* at 30 mg/Kg).

^&^Tumour volume inhibition was calculated as the nadir of the effect by the following formula: (tumour volume treated mice/tumour volume untreated mice-1) x100.

^#^Stable disease was defined as the maintenance of tumour volume for at least three weeks after the initiation of treatment.

$ Partial response was defined as the reduction of tumour, after the initiation of treatment, maintained for at least three weeks.

# The percentage of body weight loss was calculated after the initiation of treatment.
