# Supplementary material for: Focal adhesion kinase inhibitor TAE226 combined with Sorafenib slows down hepatocellular carcinoma by multiple epigenetic effects
Source: J Exp Clin Cancer Res. 2021 Nov 16;40:364. doi: 10.1186/s13046-021-02154-8 (PMC8597092; doi:10.1186/s13046-021-02154-8)
Supplement: Supplementary file 3 — Additional file 3: Table S3. Common up- and down-regulated genes. [file 13046_2021_2154_MOESM3_ESM.docx]

**Table S3. Common down- and up-regulated genes.**

| Up-regulated genes | Down-regulated genes |
| --- | --- |
| *APC* | *BCL2* |
| *CARD17* | *BHLHE40* |
| *CDK17* | *CA9* |
| *CGRRF1* | *CD70* |
| *ERBB3* | *CDC42* |
| *FBP1* | *CENPA* |
| *FOS* | *COL1A1* |
| *FRZB* | *COL4A2* |
| *LCN2* | *DLG3* |
| *RIPK1* | *EGR3* |
| *ROCK1* | *GRB7* |
| *SARS* | *LCK* |
| *TLR3* | *MAP2K2* |
|  | *MARS* |
|  | *MMP1* |
|  | *MMP3* |
|  | *NR4A1* |
|  | *OXCT1* |
|  | *PFKP* |
|  | *PRAME* |
|  | *RAC2* |
|  | *RAD51* |
|  | *RASGRF1* |
|  | *RELA* |
|  | *SLC2A3* |
|  | *TIE1* |
|  | *TLR4* |
