# Supplementary material for: Focal adhesion kinase inhibitor TAE226 combined with Sorafenib slows down hepatocellular carcinoma by multiple epigenetic effects
Source: J Exp Clin Cancer Res. 2021 Nov 16;40:364. doi: 10.1186/s13046-021-02154-8 (PMC8597092; doi:10.1186/s13046-021-02154-8)
Supplement: Supplementary file 5 — Additional file 5: Table S5. GO annotations. [file 13046_2021_2154_MOESM5_ESM.docx]

**Table S5. GO annotations.**

|  | GO term | Description | Count in Network | Strength | p |
| --- | --- | --- | --- | --- | --- |
| 1 | [GO:0000127](http://amigo.geneontology.org/amigo/term/GO:0000127) | transcription factor TFIIIC complex | 3 of 6 | 1.93 | 0.00018 |
| 2 | [GO:0016581](http://amigo.geneontology.org/amigo/term/GO:0016581) | NuRD complex | 4 of 14 | 1.68 | 5.28e-05 |
| 3 | [GO:0031595](http://amigo.geneontology.org/amigo/term/GO:0031595) | nuclear proteasome complex | 2 of 7 | 1.68 | 0.0091 |
| 4 | [GO:0008540](http://amigo.geneontology.org/amigo/term/GO:0008540) | proteasome regulatory particle, base subcomplex | 2 of 9 | 1.57 | 0.0126 |
| 5 | [GO:0030877](http://amigo.geneontology.org/amigo/term/GO:0030877) | beta-catenin destruction complex | 2 of 10 | 1.53 | 0.0148 |
| 6 | [GO:0005838](http://amigo.geneontology.org/amigo/term/GO:0005838) | proteasome regulatory particle | 4 of 21 | 1.51 | 0.00015 |
| 7 | [GO:0031597](http://amigo.geneontology.org/amigo/term/GO:0031597) | cytosolic proteasome complex | 2 of 11 | 1.49 | 0.0171 |
| 8 | [GO:0016580](http://amigo.geneontology.org/amigo/term/GO:0016580) | Sin3 complex | 2 of 11 | 1.49 | 0.0171 |
| 9 | [GO:0022627](http://amigo.geneontology.org/amigo/term/GO:0022627) | cytosolic small ribosomal subunit | 5 of 38 | 1.35 | 6.74e-05 |
| 10 | [GO:0033276](http://amigo.geneontology.org/amigo/term/GO:0033276) | transcription factor TFTC complex | 2 of 15 | 1.35 | 0.0270 |
