# Supplementary figures and images for: Focal adhesion kinase inhibitor TAE226 combined with Sorafenib slows down hepatocellular carcinoma by multiple epigenetic effects
Source: J Exp Clin Cancer Res. 2021 Nov 16;40:364. doi: 10.1186/s13046-021-02154-8 (PMC8597092; doi:10.1186/s13046-021-02154-8)

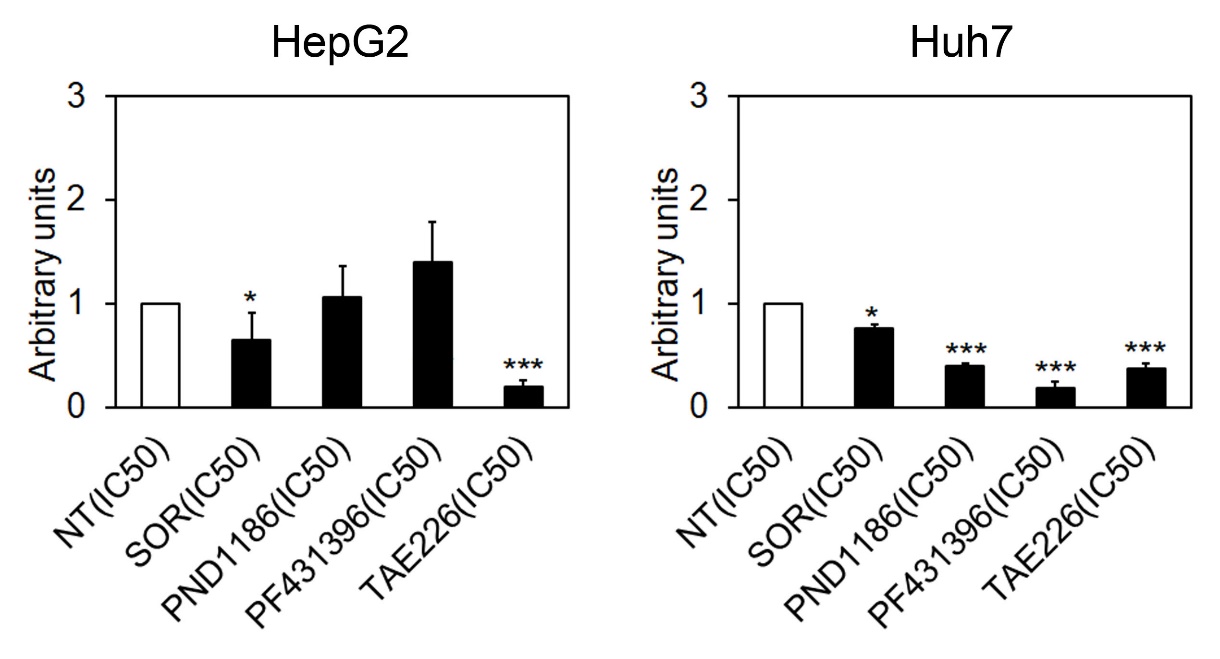

Supplement: Supplementary file 6 — Additional file 6: Figure S1. FAK phosphorylation in HCC cells after IC50 values of treatments. Quantitative analysis of pTyr397FAK expression after 48 h of treatment with the different drugs, in HepG2 and Huh7 cells. Values are the mean arbitrary units ± SD of at least three independent experiments. Data were analysed by 2-tailed Student’s t test. *p < 0.05; ***p < 0.001 vs. NT cells. [file 13046_2021_2154_MOESM6_ESM.docx]

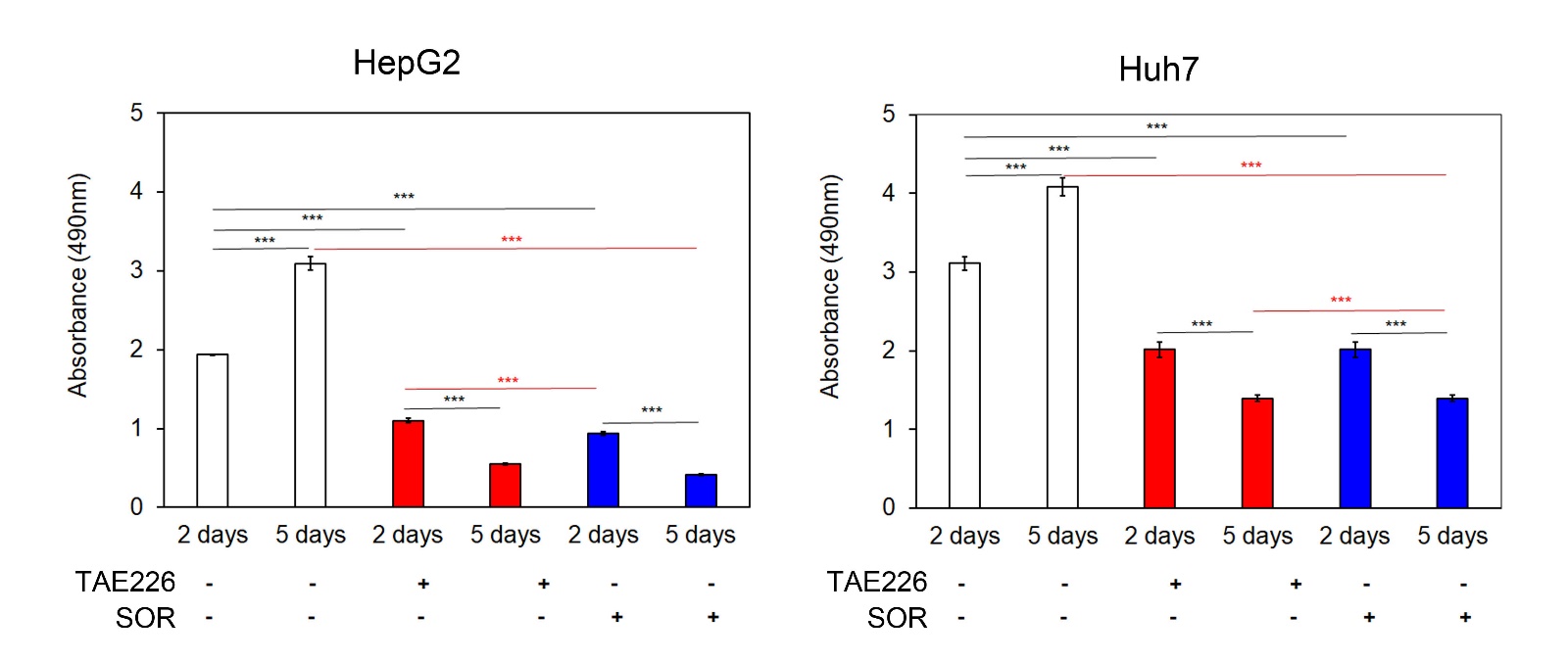

Supplement: Supplementary file 7 — Additional file 7: Figure S2. Evaluation of the IC50 values of TAE226 and SOR on HCC cell viability after 2 and 5 days. Cell viability, measured by XTT assay, after 2 and 5 days from treatment with TAE226 or SOR, in HepG2 and Huh7 cells. Values are the mean OD ± SD of three independent experiments repeated at least in duplicate. Data were analysed by ANOVA. ***p < 0.001. [file 13046_2021_2154_MOESM7_ESM.docx]

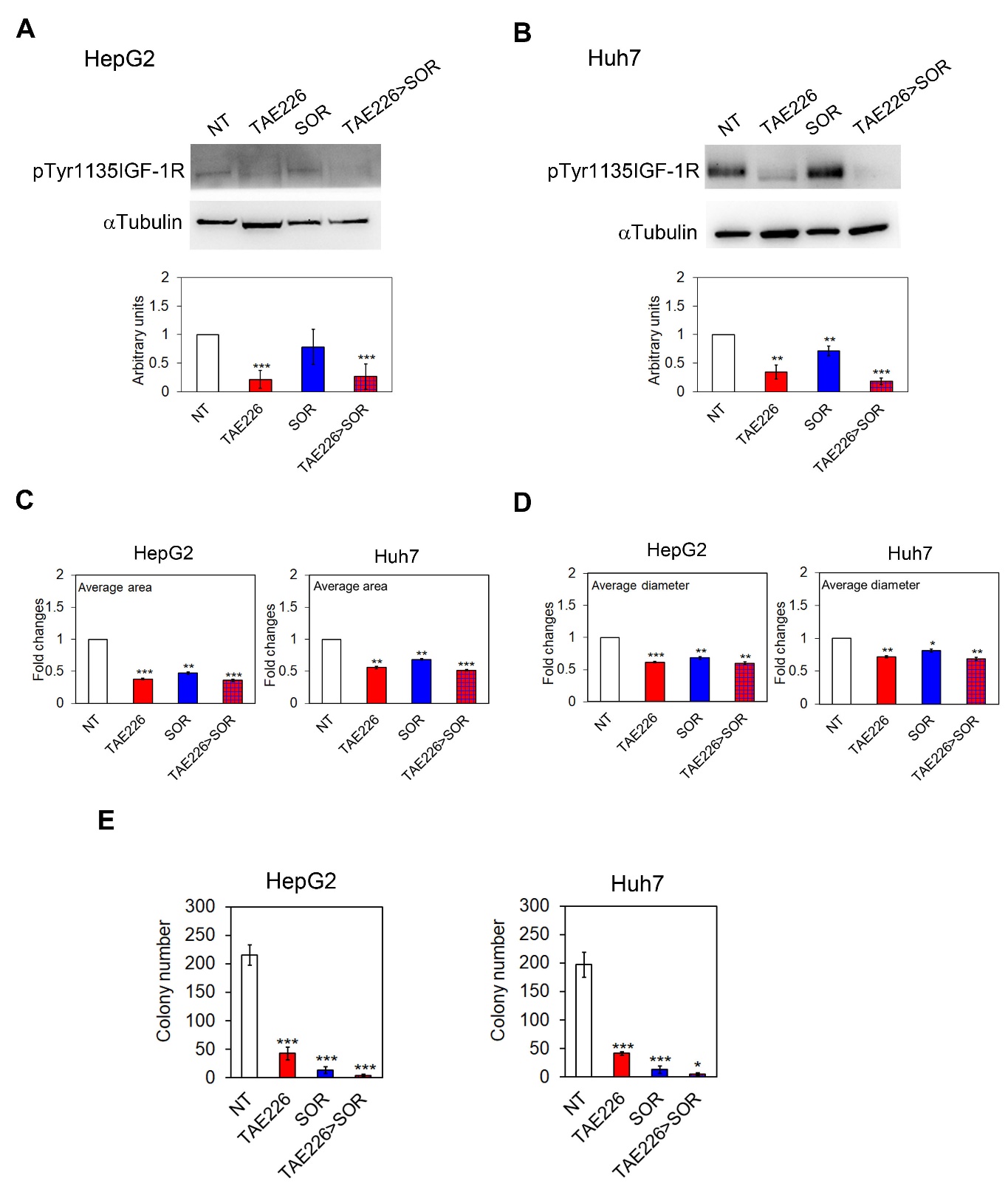

Supplement: Supplementary file 8 — Additional file 8: Figure S3. IGF-1R in HCC cells after treatment with TAE226 > SOR compared to single treatments. Representative immunoblot and quantitative analysis of phosphorylated IGF-1R after 48 h from treatment with the different drugs, in HepG2 (A) and Huh7 cells (B). αTubulin served as loading control. Values are the mean arbitrary units ± SD of at least three independent experiments. Data were analysed by 2-tailed Student’s t test. **p < 0.01; ***p < 0.001 vs. NT. Endpoints graphs representing average area (C), and diameter (D) of TS after 48 h of treatment expressed as fold induction. Data are expressed as mean ± SD of two independent experiments repeated in triplicate and were analysed by 2-tailed Student’s t test. *p < 0.05; **p < 0.01; ***p < 0.001 vs. NT. (E) Histograms of colony formation assay performed in HCC cells after treatments. Values are the mean ± SD of at least three independent experiments. Data were analysed by 2-tailed Student’s t test. *p < 0.05; ***p < 0.001 vs. NT. [file 13046_2021_2154_MOESM8_ESM.docx]

**
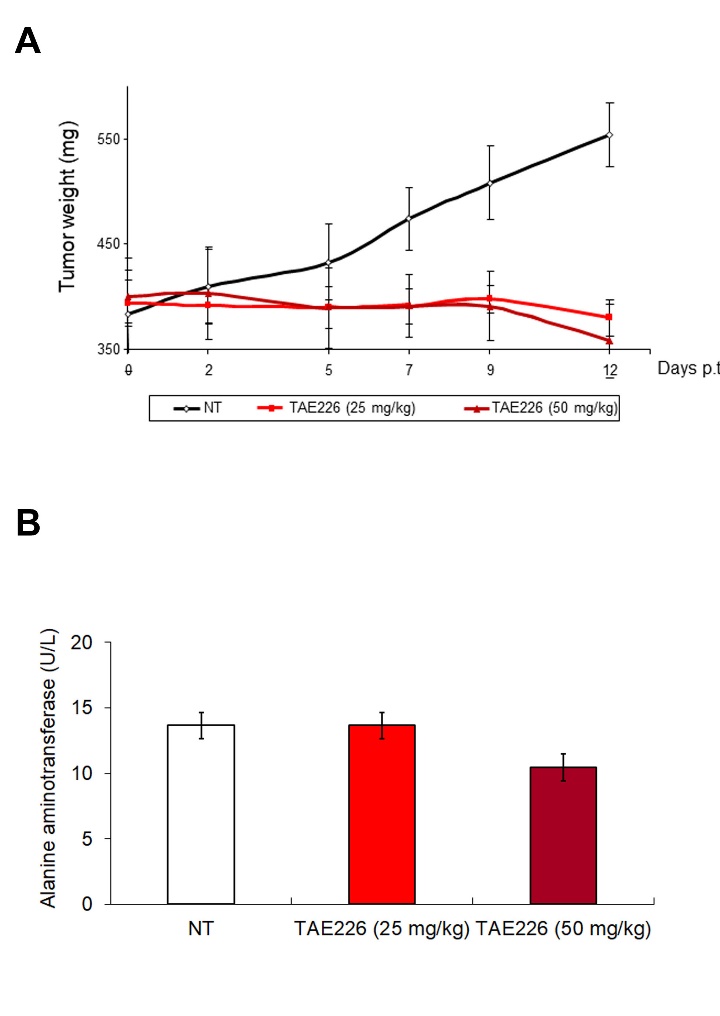
**

Supplement: Supplementary file 9 — Additional file 9: Figure S4. Pilot study for quantitative setting of TAE226 concentration in a heterotopic HepG2 xenograft model of HCC. (A) Quantitative analysis of tumour weight at various time points in NT animals or after TAE226 treatments. P values were calculated at day 12 using an unpaired two-tailed t-test, between treated and untreated tumours. **** p<0.0001. (B) Quantitative analysis of serum liver enzyme alanine aminotransferase (ALT) levels, expressed as U/L, in NT animals or after TAE226 treatments. [file 13046_2021_2154_MOESM9_ESM.docx]

**
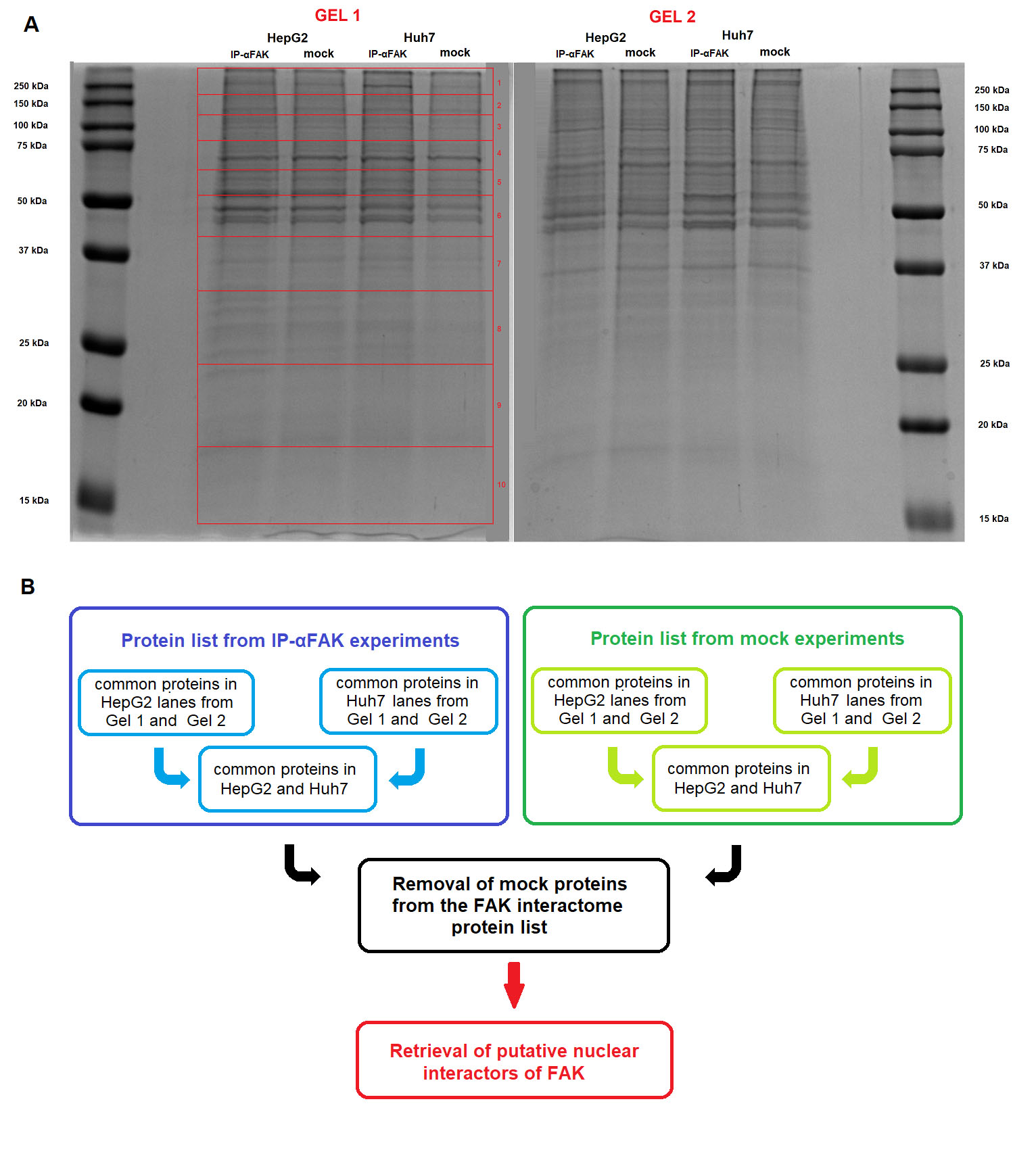
**

Supplement: Supplementary file 10 — Additional file 10: Figure S5. Analysis of FAK nuclear interaction with specific epigenetics regulators. (A) Coomassie-stained 1D SDS-PAGE gels used for the in-gel digestions of nuclear immunoprecipitates of FAK in HCC cells. The photographs showed gel lanes excision into 10 bands for mass spectrometry characterization of FAK nuclear interactors. (B) Pipeline employed for the analysis of proteomics data. [file 13046_2021_2154_MOESM10_ESM.docx]

**
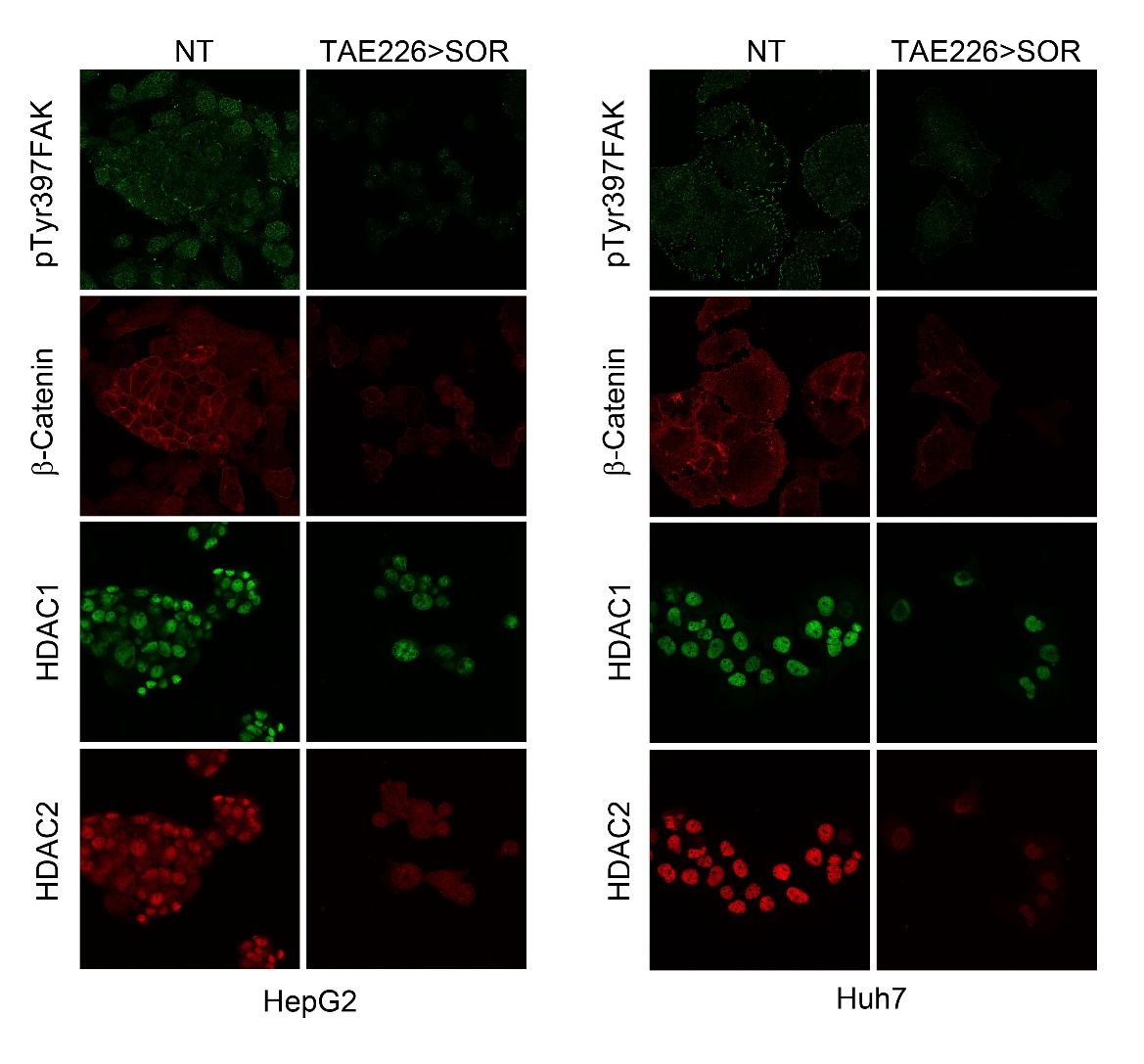
**

Supplement: Supplementary file 11 — Additional file 11: Figure S6. pTyr397FAK nuclear interactors before and after treatment with TAE226 > SOR. Representative images of immunofluorescence for pTyr397FAK, β-Catenin, HDAC1 and HDAC2 in HepG2 and Huh7 cells NT and after TAE226 > SOR treatment. 60X Magnification. [file 13046_2021_2154_MOESM11_ESM.docx]
