# Supplementary figures and images for: Correction to: Focal adhesion kinase inhibitor TAE226 combined with Sorafenib slows down hepatocellular carcinoma by multiple epigenetic effects
Source: J Exp Clin Cancer Res. 2022 Jan 27;41:40. doi: 10.1186/s13046-022-02247-y (PMC8793270; doi:10.1186/s13046-022-02247-y)

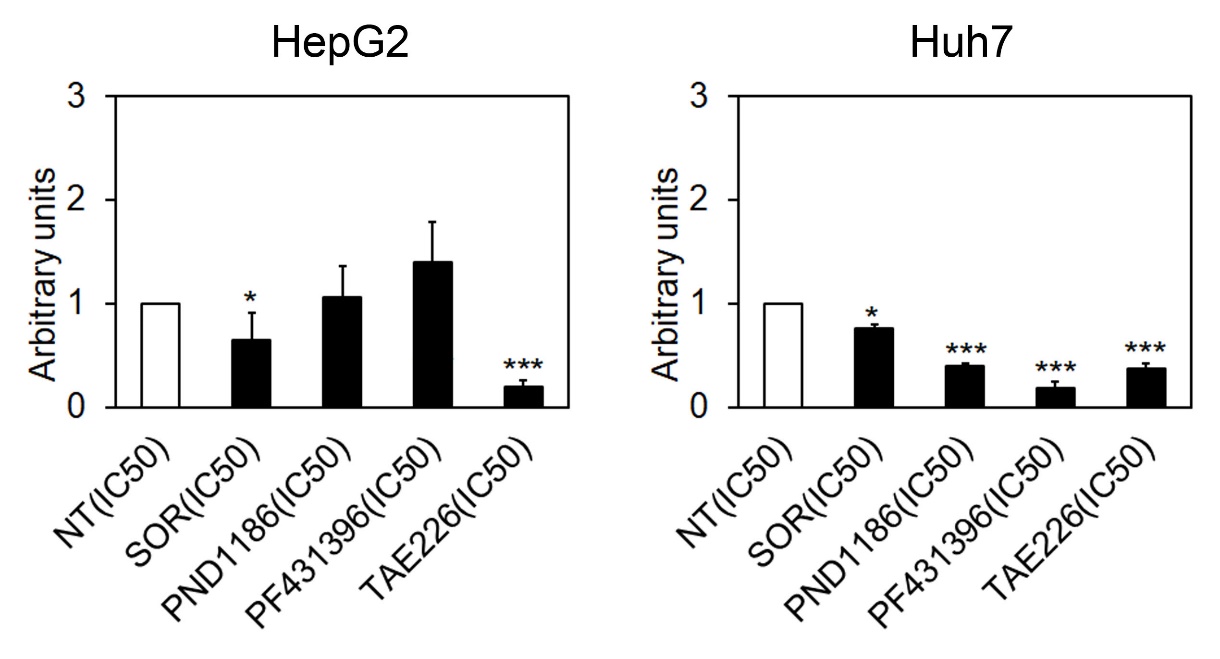

Supplement: Supplementary file 1 — Additional file 6. [file 13046_2022_2247_MOESM1_ESM.docx]

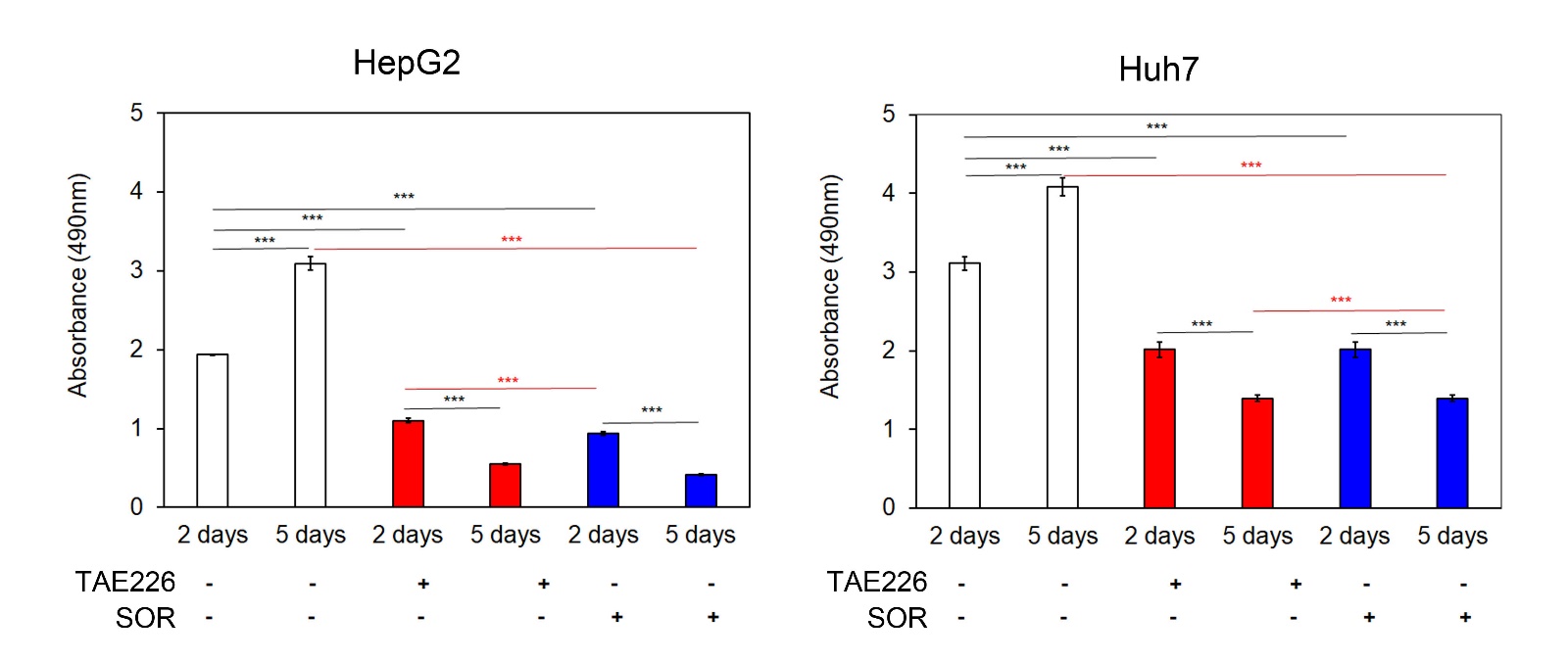

Supplement: Supplementary file 2 — Additional file 7. [file 13046_2022_2247_MOESM2_ESM.docx]

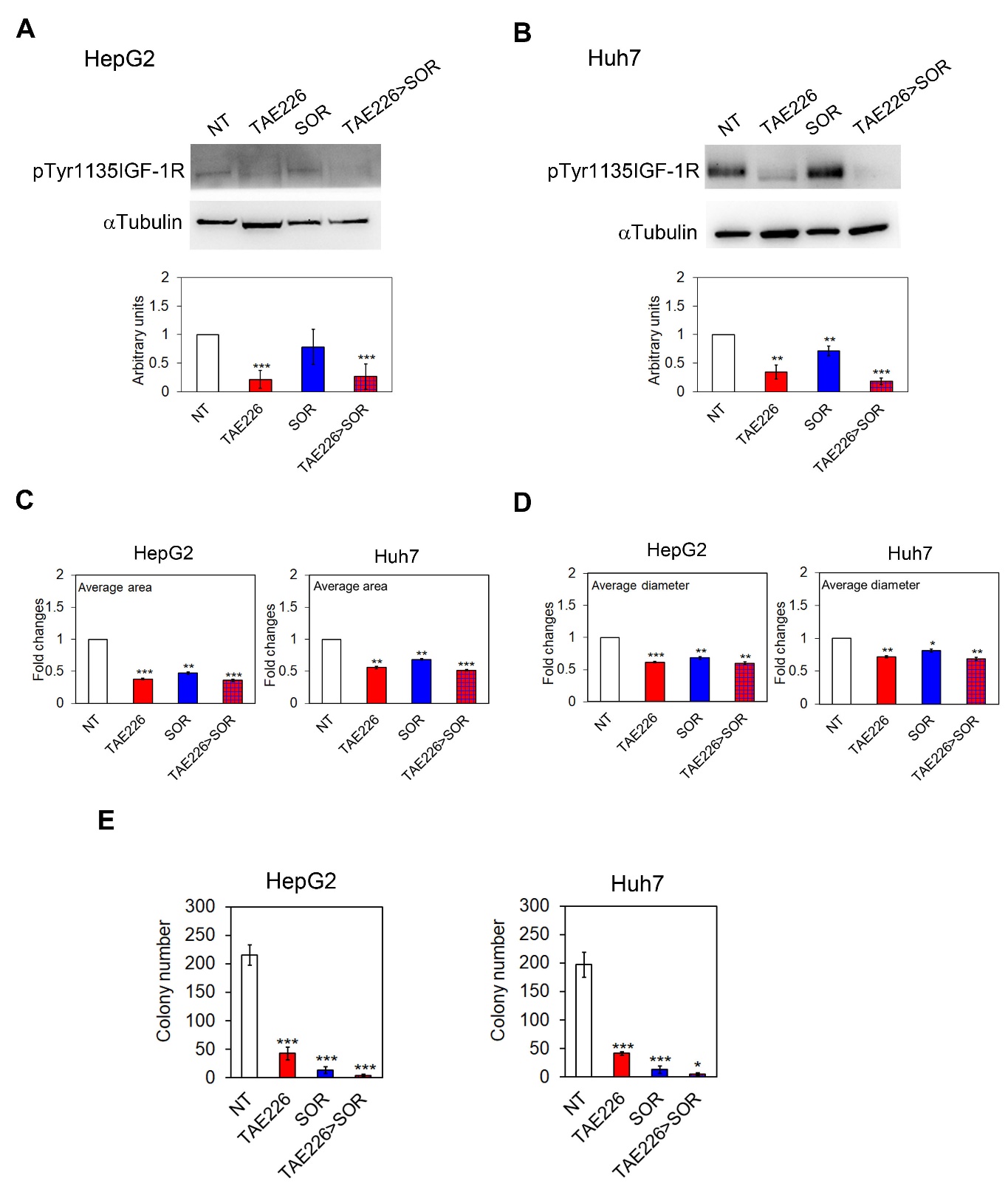

Supplement: Supplementary file 3 — Additional file 8. [file 13046_2022_2247_MOESM3_ESM.docx]
